# A novel cupulate seed plant, *Xadzigacalix quatsinoensis* gen. et sp. nov., provides new insight into the Mesozoic radiation of gymnosperms

**DOI:** 10.1002/ajb2.1853

**Published:** 2022-06-14

**Authors:** Ashley A. Klymiuk, Gar W. Rothwell, Ruth A. Stockey

**Affiliations:** ^1^ Department of Biological Sciences University of Manitoba Winnipeg Manitoba R3T 2N2 Canada; ^2^ Gantz Family Collections Center, Field Museum, 1400 S Lake Shore Drive Chicago IL 60605 USA; ^3^ Department of Botany and Plant Pathology Oregon State University Corvallis OR 97331−2902 USA; ^4^ Department of Environmental and Plant Biology 317 Porter Hall, Ohio University Athens OH 45701 USA

**Keywords:** anthophyte, Apple Bay, Cretaceous, cupule, Doyleales, Gnetales, gnetophytes, pteridosperm, seed plant phylogeny

## Abstract

**Premise:**

Anatomically preserved evidence for a novel clade of gymnosperms emphasizes diversity of seed plants immediately prior to the appearance of angiosperm fossils in the paleontological record.

**Methods:**

Cupulate seeds from the Early Cretaceous Apple Bay locality (Vancouver Island) are described from serial cellulose acetate peels and three‐dimensional reconstruction. Phylogenetic context is assessed through the comparative analysis of gymnosperm seed producing fructifications and maximum parsimony analysis of a revised morphological data set for seed plant phylogeny.

**Results:**

*Xadzigacalix quatsinoensis* gen. et sp. nov. is characterized by an orthotropous ovule with an elongated micropyle and complex integument, enclosed within a radial cupule. The micropylar canal is elongated; and the nucellus extends into the micropyle to seal the post pollination ovule. Except at the apex of the micropyle, the seed is completely enclosed by a parenchymatous cupule with ca. 20 axially elongated secretory ducts. The cupulate seed is produced upon a triangular woody stele, consisting of a parenchymatous pith surrounded by radially aligned tracheids. The stele produces three short terete traces that terminate within the base of the cupule as transfusion tissue at the seed chalaza.

**Conclusions:**

Organography, vascularization, nature of the integument and nucellus, and configuration of the micropylar canal distinguish *Xadzigacalix quatsinoensis* from all other gymnosperm clades. Cladistic analyses suggest the new plant may have affinities with gnetophytes or angiosperms. These results are complemented with a critical re‐evaluation of ovulate structures for Mesozoic gymnosperms, providing new insight into plant diversity immediately antecedent to the explosive diversification of flowering plants.

The late Mesozoic was a time of profound floristic change with the origin, rapid diversification and rise to dominance of angiosperms and the appearance of many seed plant clades (e.g., Crane, 1987; Wing et al., 1993, 2009; Doyle, 2014; Herendeen et al., 2017; Coiro et al., 2019). Although flowering plants became significant components of terrestrial communities throughout the Cretaceous (Crane, 1987; Wolf and Upchurch, 1987; Wing et al., 2009; Leslie et al., 2018; Condamine et al., 2018), the picture of biotic change in the late Mesozoic is not simply one of exclusion or replacement: Pinaceous and cupressaceous conifers reached the zenith of their diversity in the northern hemisphere (Miller, 1976; Klymiuk and Stockey, 2012; Ryberg et al., 2012; Herrera et al., 2016; Falcon‐Lang et al., 2016; Smith et al., 2017; Leslie et al., 2018; Rothwell et al., 2020); gnetophytes flourished and then declined (Crane and Lidgard, 1989; Friis et al., 2007, 2009, 2013, 2019a; Mendes et al., 2008, 2010, 2012, 2020; Rydin and Friis, 2010); and even leptosporangiate ferns enjoyed explosive evolutionary radiations (Lovis, 1977; Rothwell, 1987). While some gymnospermous clades such as the Bennettitales, which had been major components of Mesozoic landscapes, did decline and suffer extinction during the Cretaceous, others such as the Doyleales (Stockey and Rothwell, 2009, 2016; Shi et al., 2016, 2021) first appear in this interval.

Our capacity to recognize novel clades, such as the doylealean plants, owes significantly to the availability of anatomically preserved specimens. Although only a minority of plant fossils are preserved in three dimensions at a cellular level of detail, such assemblages yield a disproportionately large percentage of information about the diversity, growth, development, and reproductive biology of extinct plants. This assertion is particularly true for the Lower Cretaceous (Valanginian) Apple Bay assemblage of northern Vancouver Island. This paleobotanical *konservat lagerstätte* offers a unique window into vascular plant diversity immediately antecedent to the appearance of angiosperms (sensu Bateman, 2020) in the fossil record. Two decades of collecting efforts at Apple Bay have yielded fossil fungi (Smith et al., 2004; Bronson et al., 2013), lichens (Matsunaga et al., 2013), liverworts and mosses (Steenbock et al., 2011; Shelton et al., 2015, 2016; Tomescu, 2016; Bippus et al., 2017; Savoretti et al., 2018), lycopodialean and selaginellalean lycophytes (Stockey and Rothwell, 2006), *Equisetum* (Stanich et al., 2009), several families of leptosporangiate ferns (Smith et al., 2003; Hernandez‐Castillo et al., 2006; Little et al., 2006; Rothwell and Stockey, 2006; Stockey et al., 2006; Vavrek et al., 2006; Jud et al., 2008), conifers (Stockey and Wiebe, 2008; Klymiuk and Stockey, 2012; Atkinson et al., 2014a, 2014b; Stockey et al., 2018), gnetophytes (Rothwell and Stockey, 2013), and other enigmatic gymnosperms (Rothwell et al., 2009; Rothwell and Stockey, 2010, 2016; Stockey and Rothwell, 2009; Ray et al., 2014). The Apple Bay assemblage thus offers an unparalleled census of plant diversity during this pivotal interval of Earth history.

In this study, we describe a novel uni‐ovulate cupulate gymnosperm fructification from the Apple Bay assemblage. These fossils, described herein as *Xadzigacalix quatsinoensis* gen. et sp. nov., do not conform to any currently recognized clade of seed plants and thus illustrate yet another unexpected facet of Cretaceous gymnosperm diversity. This discovery emphasizes that anatomically preserved lagerstätte assemblages continue to significantly expand our appreciation for the complexity of gymnosperm‐dominated assemblages immediately before the radiation of flowering plants.

## MATERIALS AND METHODS

### Geologic and depositional setting

Specimens presented here are three‐dimensionally preserved at a cellular level of detail within semi‐sideritic, marine carbonate concretions (Gierlowski‐Kordesch et al., 2021) collected from the Apple Bay locality of northern Vancouver Island, British Columbia (50°36′21″N, 127°39′25″W; UTM 9U WG 951068). The locality comprises a 6.2‐m‐thick section of 20 sedimentary beds, 13 of which are fossiliferous. The beds crop out as a rocky beach on the north shore of Holberg Inlet and are composed predominantly of clast‐supported cryptically bioturbated (sensu Pemberton et al., 2008) sandstone cemented with CaCO_3_. Sandy, stacked amalgamated beds like those at Apple Bay form on delta fronts under hyperpycnal conditions; when associated with seasonal storms, they often contain significant plant debris (MacEachern et al., 2005; Gierlowski‐Kordesch et al., 2021). Taxa preserved at Apple Bay represent a diverse assemblage of plants, including moss and liverwort gametophytes (Tomescu, 2016), fungi (Smith et al., 2004; Bronson et al., 2013), and dipteridaceous ferns (Stockey et al., 2006) consistent with a forest‐floor flora marginal to a river or stream, constituents of which were periodically swept into the marine basin during storm events (Klymiuk and Stockey, 2012). The Apple Bay locality represents one of several sedimentary basins associated with the Wrangellia Terrane (Muller et al., 1974; Hammack et al., 1994) and has been interpreted as a Lower Cretaceous Longarm Formation equivalent (Jeletzky, 1976; Haggart, 1994; Nixon et al., 2011) of possible Barremian age (Sweet, 2000). Oxygen isotope analyses indicate the locality is ~136 Ma, corresponding to the mid‐late Valanginian (D. R. Gröcke, Durham University, personal communication, 2010), and therefore probably represents the zenith of pre‐angiosperm floral diversity (Herendeen, 2017; Coiro et al., 2019).

### Specimen preparation

Concretions containing the specimens were sectioned into ~0.75‐cm‐thick wafers, exposing two specimens in oblique transverse section (UAPC‐ALTA P15375 C_bot_, D, and P16474 B_top_), and one specimen in oblique longitudinal section (P15280 B_top_). Using the cellulose acetate peel technique (Joy, 1956), we prepared serial transverse and longitudinal sections of all specimens in their entirety. Specimen peels were permanently mounted on glass slides, using xylene‐soluble Eukitt (O. Kindler GmbH, Freiberg, Germany) mounting medium. Photomicrographs were captured with a Powerphase (Phase One, Copenhagen, Denmark) digital scanning camera mounted on a Leitz Aristophot bellows camera and Zeiss WL compound microscope. Images were processed with Photoshop CS5 (Adobe, San Jose, CA, USA); image processing involved minor color and contrast adjustments applied globally. Three‐dimensional reconstructions were constructed in Avizo v5.1 (FEI Visualization Sciences Group, Hillsboro, OR, USA). Specimens are deposited in the University of Alberta Paleobotanical Collection (UAPC‐ALTA), in Edmonton, Alberta, Canada.

### Phylogenetic analyses

Phylogenetic relationships of the new taxon were assessed with a morphological matrix (Appendix S1) of 107 characters and 42 taxa. This matrix is a revision and expansion (Appendix S2) of that previously published by Rothwell and Stockey (2016), and includes character concepts originally developed by Rothwell and Serbet (1994), Hilton and Bateman (2006), and Doyle (2006, 2008). Tree searches were performed under the parsimony ratchet perturbation algorithm (Nixon, 1999), used in the program TNT (Goloboff et al., 2008) spawned through Winclada (implemented as Asado, v1.1 beta, by K. Nixon, Cornell University). If support was ambiguous, branches were collapsed. All characters were treated as nonadditive, with multistate characters unordered. In each of 10 sequential ratchet searches, ratchet perturbation sampled 10% of the parsimony‐informative characters through 10,000 iterations per replication, with 10 trees held per iteration, and a random constraint level of 12 (Goloboff et al., 2018). Only the semi‐strict consensus phylogeny is reported; bootstrap (Felsenstein, 1985) values were generated from 1000 replicates, with 10 trees held for each of 100 multiple tree‐bisection‐reconnection (TBR) search replications. The morphological matrix was also explored using PAUP* v4.0a168 (Swofford, 2003), where we employed maximum parsimony heuristic searching with characters unordered and equally weighted; gaps were treated as missing, and multistate taxa were interpreted as polymorphisms. Starting trees were obtained by random stepwise addition for 10,000 replicates with one tree held at each step. Branch swapping was performed by TBR with a reconnection limit of 8. Trees were unrooted, and branches were collapsed to polytomies if the branch length was zero. Bootstrap values were generated from 100 bootstrap replicates with one tree held for each of 100 TBR heuristic search replications with reconnection limit of 8.

## RESULTS

### Systematics

Class Spermatopsida

Order Indet.

Family Indet.

**Generic diagnosis**: *Xadzigacalix* Klymiuk, Rothwell et Stockey gen. nov.

Gymnospermous plants bearing radial uni‐ovulate cupulate seeds terminally on woody stems. Cylindrical stele dividing into three terete vascular bundles within base of cupule, terminating as transfusion tissue at chalaza of ovule. Ovule orthotropous, radially symmetrical; integument complex, unvascularized, forming elongated micropylar canal. Nucellus with thickened cuticle; apex solid, cellular; attached to integument only at chalaza. Ovule surrounded by radial cupule except at protruding micropylar canal, attached only at chalaza.

**Etymology**: *Xadzigacalix* is compounded of Latin (calix = chalice), and transliterated Kwak̓wala (xa̱dziga = plant resin), the language of the Kwakwaka'wakw nations of the northwestern coastline of North America. The Apple Bay locality, from which the new genus is described, occurs within traditional and unceded territory of these Indigenous peoples. The name *Xadzigacalix* denotes the morphology and histology of the cupule. (pronunciation, styled after the Oxford English Dictionary:/sʧædz:ˌi:'gʌ:keɪ:'lɪks/; styled phonetically: chutdz‐i‐guh kay‐liks’\).

**Type species**: *Xadzigacalix quatsinoensis* Klymiuk, Rothwell et Stockey sp. nov.

**Specific diagnosis**: Cupule ellipsoidal, ca. 5 mm long by 3 mm diameter, glabrous, composed of two zones of parenchyma. Outer parenchyma zone of isodiametric to flattened cells 3–20 µm diameter, containing longitudinal secretory ducts up to 100 × 200 µm diameter. Ducts lined with uniseriate epithelium of secretory cells. Inner parenchyma zone of radially elongated cells 25 µm diameter grading to isodiametric cells 45–60 µm diameter, forming weakly organized palisade layer. Epidermis of abaxial/external surface with zones of radially aligned parenchyma. Ovule tetrahedral, 1.5–1.6 mm wide at midsection. Integument of three layers; outermost 45–75 µm thick composed of thick‐walled parenchyma; middle 95–100 µm thick composed of palisade sclerenchyma; innermost 50 µm thick composed of isodiametric sclerenchyma. Nucellus without thickened cuticle, expanding into micropyle at maturity, containing megagametophyte with megaspore membrane up to 250 µm thick. Micropylar canal nearly sealed at maturity with triradiate opening reflecting three angles of ovule. Cupule at apex of triangular stele 600–700 µm wide with parenchymatous pith and radial rows of helical to scalariform tracheids 6–10 µm diameter. Stele terminating below nucellus as transfusion tracheids 10–18 µm diameter and as terete cupular strands 125–145 µm diameter below each angle of ovule.

**Etymology**: The specific epithet *quatsinoensis* refers to Quatsino Sound, Vancouver Island, British Columbia. (pronunciation:/'cwɑt:ˌsi:noʊ:'ɛn:sɪs/)

**Holotype hic designatus**: Uni‐ovulate cupule in transverse section (P15375 C_bot_ and D). Paratypes comprise less mature uni‐ovulate cupules in longitudinal (P15280 B_top_) and transverse (P16474 B_top_) sections. All specimens are prepared as cellulose acetate peels mounted on glass microscope slides; specimens are deposited in the University of Alberta Paleobotanical Collection (UAPC‐ALTA), Edmonton, Alberta, Canada.

**Type locality**: Apple Bay, Holberg Inlet, Vancouver Island, British Columbia.

**Stratigraphy**: probable Longarm Formation equivalent.

**Age**: mid‐Valanginian, ~136 Ma.

### Description of *Xadzigacalix quatsinoensis*

**Figures 1–7:**
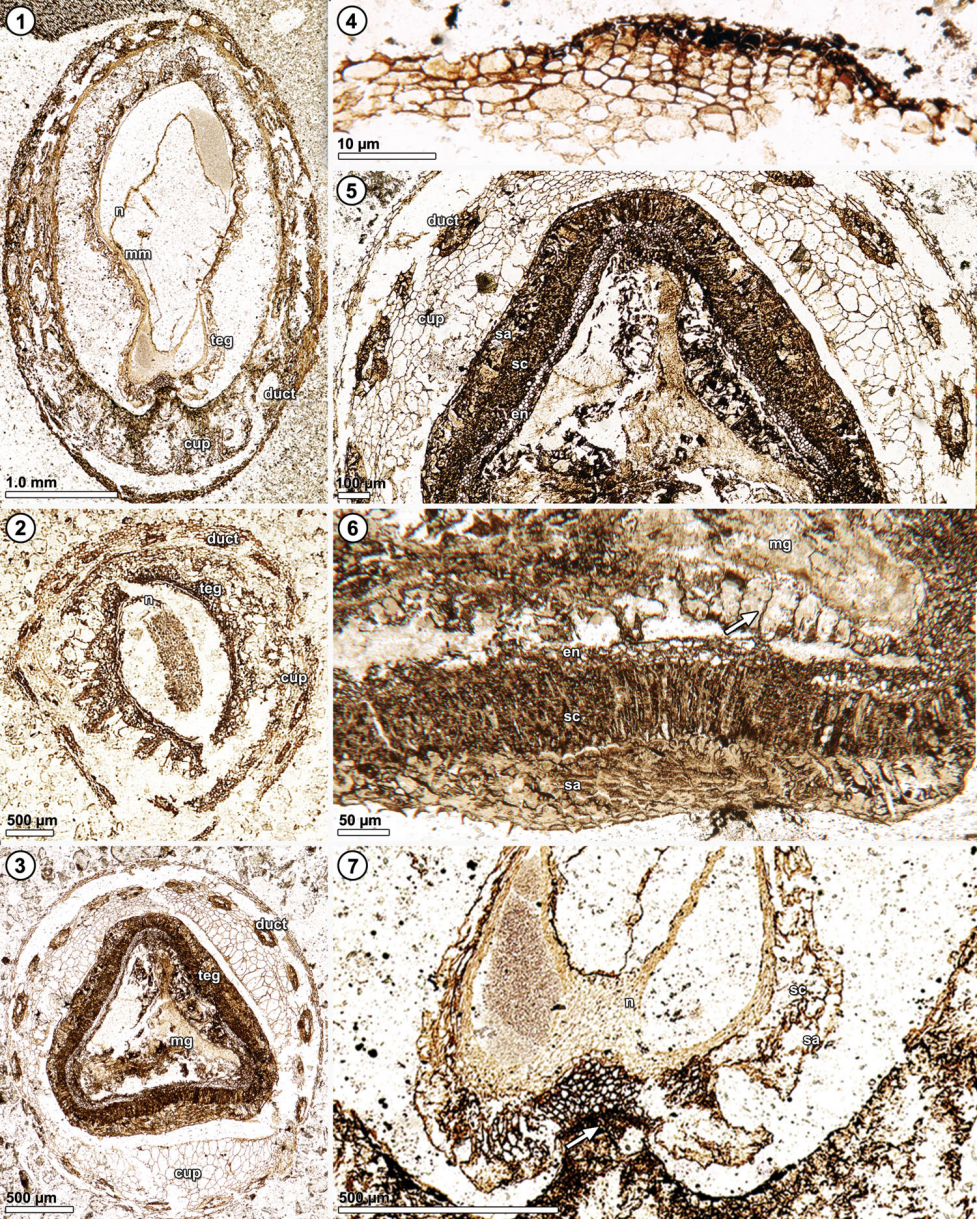
*Xadzigacalix quatsinoensis* gen. et sp. nov., development and histology of ovule and enclosing cupulate structure. **1**. Oblique longitudinal section of immature ovule basally attached to cupulate (cup) structure. Note developing integument (teg) of ovule, nucellus (n) with chalazal attachment, and megaspore membrane (mm), and secretory ducts (duct) throughout cupule. P15280 B_top_ #1. **2**. Immature ovule in transverse section, with developing integument (teg) and nucellus (n), enclosed by cupulate structure (cup) P16474 B_top_ #25. **3**. Mature seed in transverse section, with complex integument (teg) and megagametophyte (mg) tissue. Note histology of enclosing cupulate structure (cup), with outermost zone of thin‐walled isodiametric parenchyma containing secretory ducts (duct) with distinctive epithelial lining. P15375 C_bot_ #51. **4**. Serially arranged files of parenchymatous cells along exterior surface of cupulate structure P15375 C_bot_ #175. **5**. Histology of seed integument showing sarcotesta (sa), sclerotesta (sc), multiseriate endotesta (en), and histology of cupulate structure (cup), showing secretory ducts (duct) lined with darkly pigmented epithelial cells. Note histological zonation of cupule, with large, elongate parenchyma internal to zone of smaller, isodiametric parenchyma containing ducts. P15375 C_bot_ #67. **6**. Histology of seed integument (sarcotesta, sa; sclerotesta, sc; endotesta, en), prominent megaspore membrane (arrow) enclosing megagametophyte (mg). P15375 C_bot_ #4. **7**. Chalaza of immature ovule showing developing integument (Sarcotesta (sa) and sclerotesta (sc) are differentiating), basal attachment of nucellus (n), and transfusion tissue (arrow) at point of attachment with cupulate structure. P15280 B_top_ #1.

**General features**: The gymnospermous *Xadzigacalix quatsinoensis* plant bears uni‐ovulate, radial cupules terminally upon an apparently eustelic woody stem tip. Whether or not individual cupulate seeds form part of a larger fructification is unknown. Seeds are characterized by a complex integument and extended micropylar canal, and the cupule has distinctive longitudinal secretory ducts. Three specimens of this species are presently available for study (Figures 1–7, 21): a histologically mature ovule in transverse section (Figure 1–7, 21), in which the micropyle and subchalazal vascular architecture are preserved; and two histologically immature specimens, in oblique longitudinal (Figure 1–7, 21) and transverse section (Figure 1–7, 21). Together, these specimens reveal features of cupule attachment, seed and integument structure and development, and permit characterization of the nucellus. All three ovules are enclosed in ellipsoidal cupules that are circular in cross section and approximately 5 mm long and 3 mm in diameter at maturity. Cupules appear to achieve mature dimensions and histological differentiation before ovule maturation, as evidenced by the presence of an ovule at an early stage of development within a much larger cupule (Figure 1–7, 21); at maturity, the ovule entirely fills the available space within the cupule (Figure 1–7, 21). Cupules have two histological zones throughout and show small areas of secondary parenchyma formation (Figure 1–7, 21).

**Ovule**: The ovule is ca. 4.5–5.5 mm long, 3.0–3.5 mm in diameter at maturity, tetrahedral and triangular in cross section, and with each side up to 1.1–1.2 mm wide in the midregion (i.e., Figure 1–7, 21). Histologically, the ovule is characterized by a complex integument differentiated into outer sarcotesta with uniseriate epidermis, sclerotesta, and endotesta. The sarcotesta is composed of thick‐walled irregularly shaped parenchyma forming a layer 45–50 µm thick at the corners and thickening to ca. 75 µm along the sides of the ovule. The sclerotesta is 95–100 µm thick and composed of ~five to seven layers of thick‐walled sclereids in a palisade arrangement. Endotesta consists of four to six layers of smaller isodiametric sclerotic cells ca. 50 µm in diameter (Figures 1–7, 21).

**Figures 8–10:**
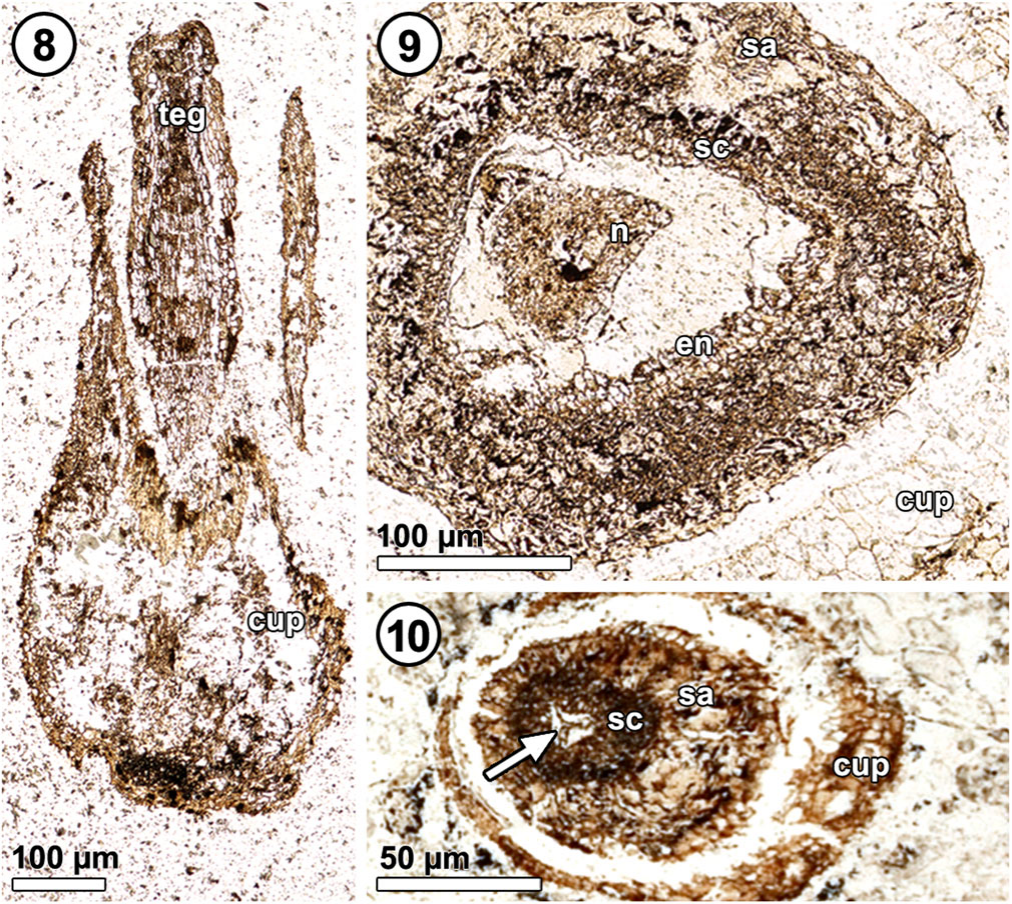
*Xadzigacalix quatsinoensis* gen. et sp. nov., cupule apex and micropyle of seed. **8**. Oblique section through micropylar canal at apex of ovule; ovule integument (teg) extends beyond cupule tissue (cup). P15280 A #1. **9**. Transverse section near ovule apex; note solid cellular nucellus (n) and integumentary layers comprising sarcotesta (sa), sclerotesta (sc), and endotesta (en). P15375 D #3. **10**. Micropylar apex showing sarcotesta (sa) and sclerotesta (sc) integumentary layers; note nucellar apex (arrow). P15375 D #60.

Apically, the ovule forms an elongate cylindrical micropyle (Figure 8–10, 30) 310–325 µm in diameter at the base (Figure 8–10, 30), that protrudes at least 100 µm beyond the cupule rim and that narrows (Figures 1–7, 8–10) to ca. 75 µm at the apex (Figure 8–10, 30). The micropylar canal also narrows distally, becoming nearly closed at its apex save for a tri‐radiate opening, ca. 15 µm wide (Figure 8–10, 30, at arrow; Appendix S3).

**Nucellus and megagametophyte**: The nucellus is attached to the integument at the chalaza and free at more distal levels (Figure 8–10, 30). It lacks the prominent outer cuticle that characterizes many other gymnospermous ovules (Figures 1–7, 21). Taphonomically altered tissue within the nucellus of the longitudinally sectioned specimen may represent cellular megagametophyte (Figure 1–7, 21). In the most mature specimen, a distinctive megaspore membrane is clearly visible (Figures 1–7, 21) and surrounded by isodiametric to rectangular cells of the nucellus that are up to 25 µm wide and 75 µm high (Figure 1–7, 21, at arrow). The apex of the nucellus shows no evidence of a pollen chamber, but instead remains solid as it projects up into the micropylar canal (Figures 8–10, 30, 8–10, 30; Appendix S3), and assumes a tri‐radiate configuration in cross section (Figure 8–10, 30). There are remnants of megagametophyte or embryonic tissue in one specimen (Figure 1–7, 21), but cellular details are incompletely preserved.

**Cupule**: The cupule almost completely surrounds and closely adheres to the seed (Figures 1–7, 21), except at the apex where the elongated micropylar tube protrudes beyond the cupule rim (Figures 8–10, 30). It is composed of two zones of thin‐walled parenchyma. The outer zone of parenchyma consists of smaller (13–20 µm diameter) cells (Figures 1–7, 21) and contains distinctive secretory ducts that extend the length of the cupule (Figure 1–7, 21). The secretory ducts are histologically distinctive (Figures 1–7, 21), appearing in cross section either as oval areas of darkened secretory cells (Figure 1–7, 21), or as elliptical rings of such cells surrounding a hollow center (Figures 1–7, 21). Ducts typically measure 100 × 200 µm at their widest (Figure 1–7, 21). Most secretory ducts originate below the level of ovule attachment to the cupule, and several extend most of the length of the cupule (Figure 14). Basal portions of the cupule have discrete areas of radially aligned secondary parenchyma cells near the periphery that are consistent with periderm initiation or wound response parenchyma (Figure 1–7, 21). Cells of the inner parenchyma zone are radially elongate (ca. 25 µm diameter), grading into larger‐diameter (45–60 µm) isodiametric cells, which together form a weakly organized palisade (Figures 1–7, 21). The interior surface of the cupule is lined by a layer of epidermal cells that are 7–10 µm wide.

**Figures 11–13:**
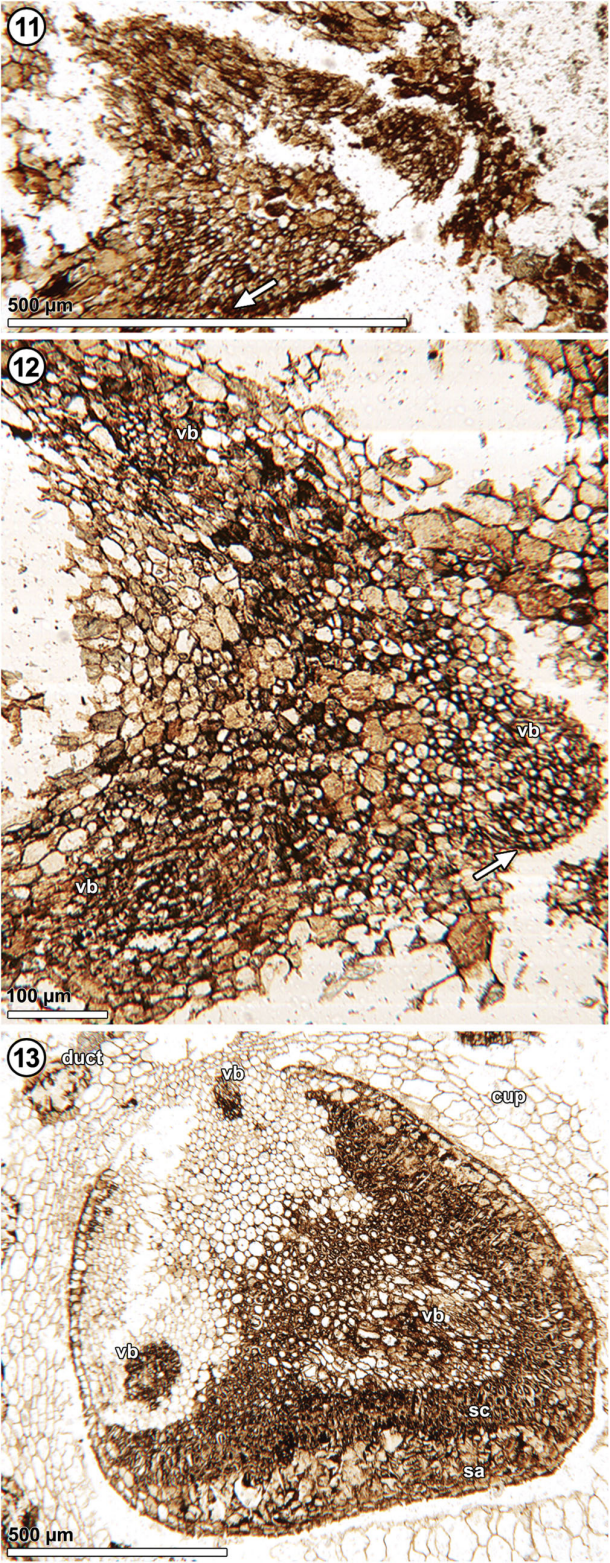
*Xadzigacalix quatsinoensis* gen. et sp. nov., chalaza and vascular architecture subtending ovule. **11**. Incomplete triangular stele at base of cupule; note radially aligned tracheids (arrow) and parenchymatous pith. P15375 C_bot_ #253. **12**. Oblique section distal to triangular stele, showing three vascular bundles (vb); note radially aligned tracheids (arrow). P15375 C_bot_ #227. **13**. Chalaza of mature seed with differentiated integumentary layers (sarcostesta, sa; sclerotesta, sc), and terete vascular bundles (vb) terminating within integument at chalaza (arrow). P15375 C_bot_ #143.

**Vascular tissue and architecture**: At the basalmost level of the fossils, there is a triangular stele (Figure 11–13, 31) that consists of a woody cylinder with a parenchymatous pith (Figures 11–13, 31, 20). There are at least six rows of radially aligned tracheids 10–14 µm in diameter. Progressing distally, the stele divides to produce three terete vascular strands, 125–145 µm in diameter, that terminate within the base of the cupule on radii that correspond to the angles of the ovule (Figures 11–13, 31, 16, 17). Tracheids of the vascular strands are narrow, 6–10 µm, and have scalariform secondary wall thickenings (Figure 19). The central region of the stele extends into the chalaza of the seed and terminates as a mixture of parenchyma cells and transfusion tracheids at the base of the nucellus (Figures 1–7, 21, 11–13, 31). Transfusion tracheids measure 10–18 µm in diameter and have helical to scalariform secondary wall thickenings (Figure 18).

**Figures 14–17:**
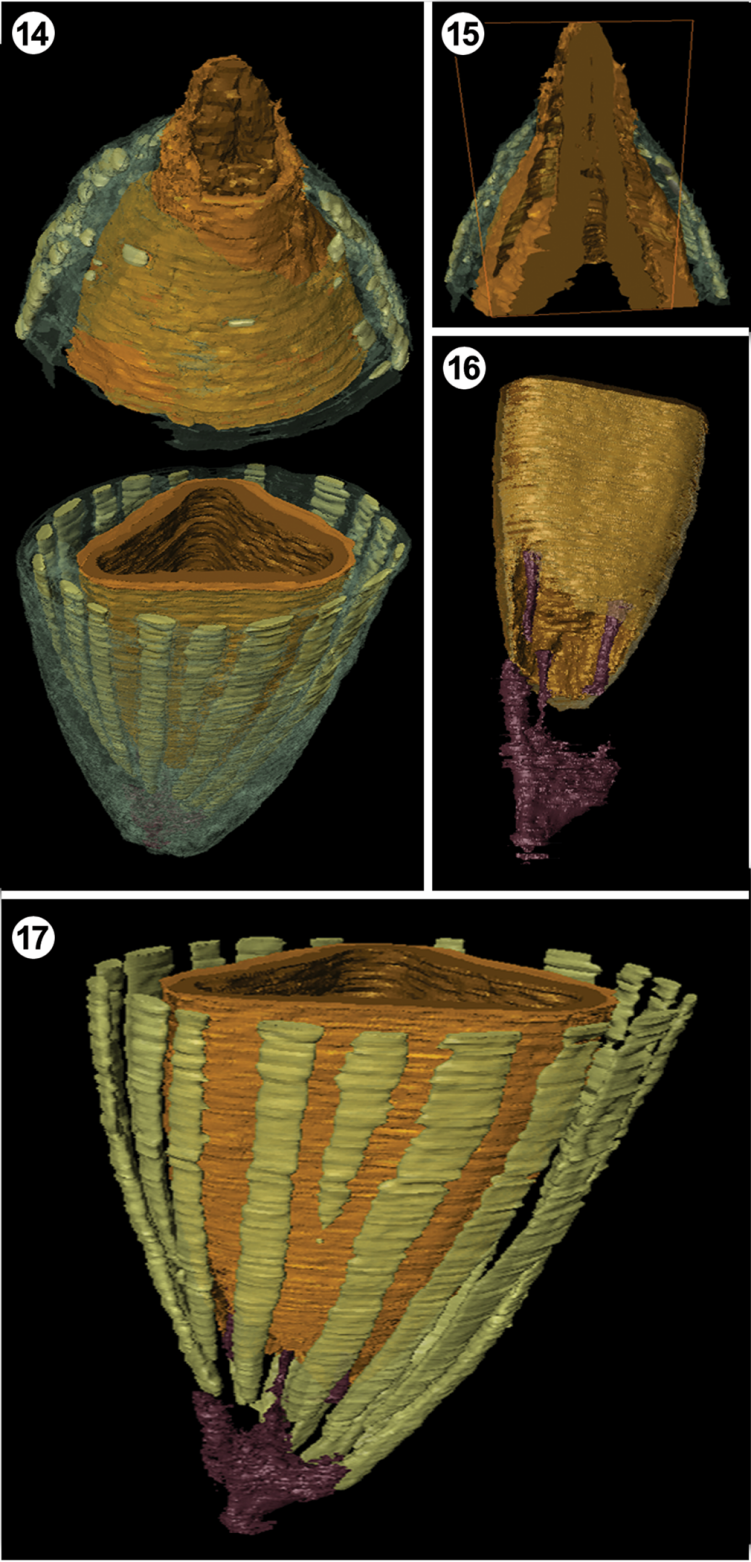
*Xadzigacalix quatsinoensis* gen. et sp. nov., three‐dimensional reconstruction. **14**. Cupulate seed reconstructed from oblique transverse sections of base, P15375 Cbot, and apex, P15375 D. Neither nucellus nor endotesta have been reconstructed; ovule tissues in reconstruction comprise sarcotesta (exterior, golden), sclerotesta (interior, dark brown); secretory ducts (light green) within cupule (translucent green) are absent from one side of specimen owing to abrasion before fossilization. **15**. Apex of ovule enclosed by cupulate structure, with digital longitudinal section illustrating extension of micropylar canal beyond cupule. P15375 D. **16**. Ovule (sarcotesta = golden, sclerotesta = brown) vascularized by three bundles produced from woody stem (puce). **17**. Arrangement of secretory ducts with respect to chalaza of ovule and vascular tissue. P15375 D.

**Figures 18–20:**
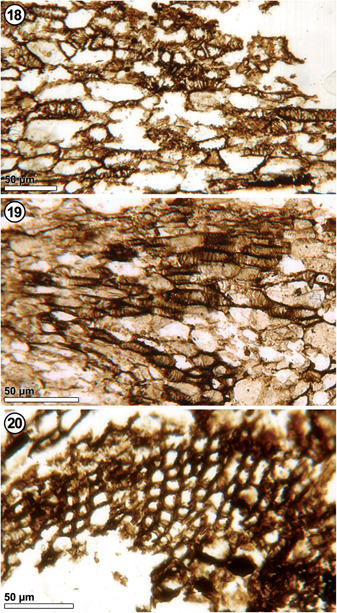
*Xadzigacalix quatsinoensis* gen. et sp. nov., vascular tissues within cupulate structure. **18**. Transfusion tracheids beneath chalaza of ovule. P15280 B_top_ #240. **19**. Tracheids with helical to scalariform thickenings. P15375 C_bot_ #248. **20**. Radially aligned tracheids forming woody cylinder at base of stele. P15280 B_top_ #206.

### Phylogenetic context of *Xadzigacalix quatsinoensis*

We performed two parallel maximum parsimony analyses of a 42‐terminal, 107‐character morphological matrix (Appendix S1) to hypothesize on the phylogenetic relationships of this novel cupulate gymnosperm. Parsimony analyses employing ratchet perturbation (Nixon, 1999) generated 60 most parsimonious tree topologies (tree length = 363, CI = 0.45, RI = 0.76); 20 most parsimonious trees were recovered with TBR heuristic searching in PAUP* (length = 404, CI = 0.51, RI = 0.77). Consensus topologies of trees generated under both search strategies are identical; therefore, we present only the semi‐strict consensus topology of trees generated with parsimony ratchet perturbation (Figure 1–7, 21).

**Figure 21:**
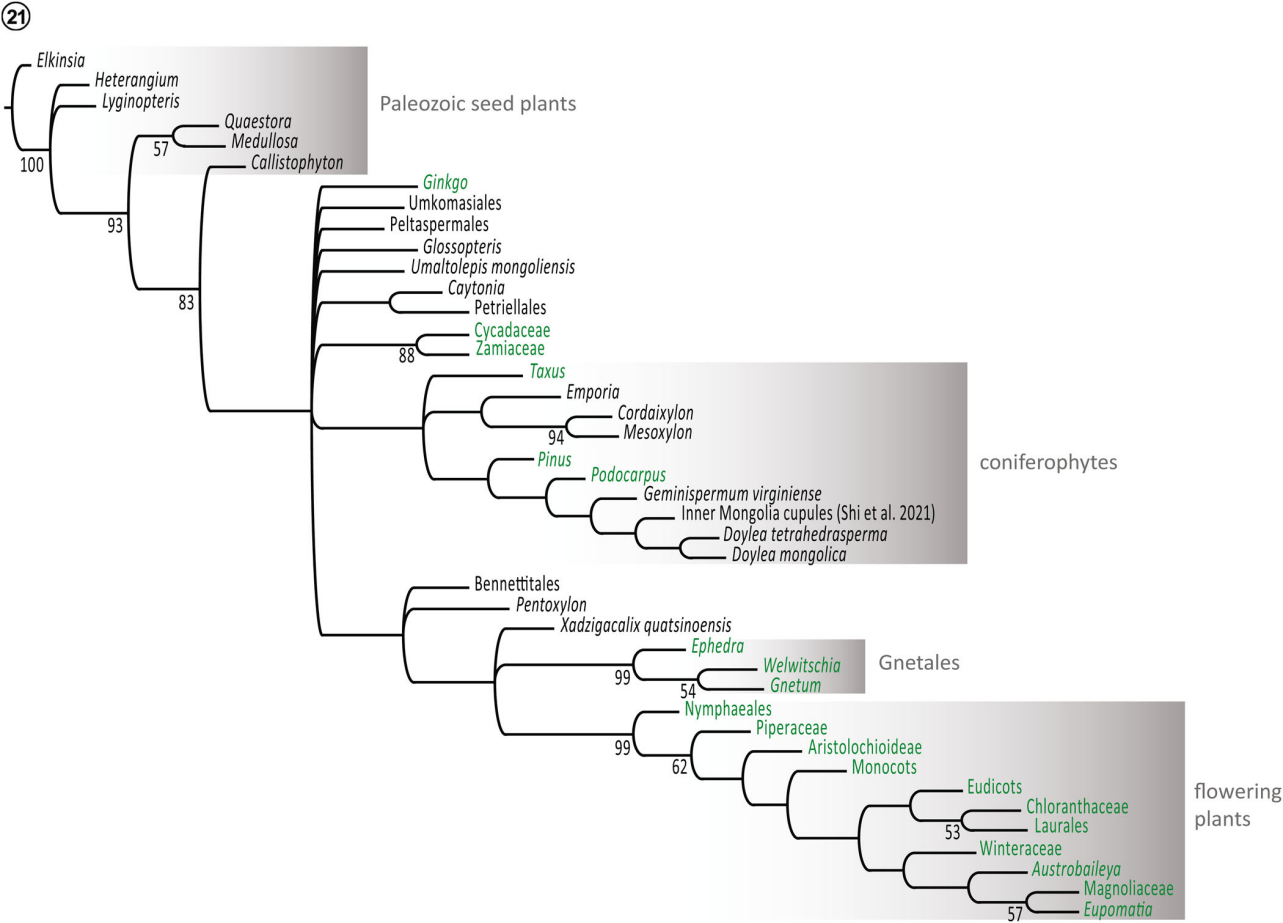
Semi‐strict consensus cladogram of 60 most parsimonious trees. Bootstrap support values >50 are reported.

In the consensus topology (Figure 1–7, 21), extinct Paleozoic seed plants constitute a basal‐grade sister to all Mesozoic and extant seed plants. Living cycadophytes (bootstrap support, BS = 88), angiosperms (BS = 99), and Gnetales (BS = 99) were recovered as monophyletic lineages with high levels of bootstrap support. Operational taxonomic units (OTUs) representing extant Taxaceae, Pinaceae, and Podocarpaceae assort within a coniferophyte clade that lacks basal resolution, but recovers the fossil taxa *Cordaixylon* Grand'Eury and *Mesoxylon* D.H. Scott et Maslen as sister taxa (BS = 94). Doylealean cupulate plants and *Geminispermum virginense* E.M. Friis, P.R. Crane et K.R. Pedersen (2019b) also assort with coniferophytes, where they form a sister clade to podocarpaceous conifers.

The second major clade of Mesozoic through modern seed plants includes the angiosperms, which are resolved as sister to a clade consisting of the gnetophytes and the new plant *Xadzigacalix quatsinoensis* (BS < 50). Bennettitalean plants + *Pentoxylon* B.P. Srivast. form a clade that is attached to the stem of the tree immediately basal to the node (BS < 50) where the angiosperms + [gnetophytes + *Xadzigacalix*] clade is attached (Figure 1–7, 21).

## DISCUSSION

### Gymnospermous seed plants of the Apple Bay lagerstätte

The Valanginian Apple Bay flora of Vancouver Island, British Columbia provides an exceptional window into the prevalence and diversity of complex seed plants in the Early Cretaceous of western North America. Conifers at Apple Bay are represented by dispersed leaves and leafy shoots (Stockey and Wiebe, 2006, 2008; Atkinson et al., 2014b), pollen cones (Sanders et al., 2004; Stockey et al., 2018), seed cones, and dispersed seeds of Cupressaceae and Pinaceae (Klymiuk and Stockey, 2012; Atkinson et al., 2014a, 2014b). Other gymnospermous plants that populate the assemblage include species of Bennettitales (i.e., *Nilssoniopteris corrugata* Ray, Rothwell et R.A. Stockey, *Williamsonia* Carruthers sp., and *Foxeoidea connatum* Rothwell et R.A. Stockey (Rothwell and Stockey, 2002, 2010; Stockey and Rothwell, 2003; Rothwell et al., 2009; Ray et al., 2014), Gnetales (i.e., *Protoephedrites eamesii* Rothwell et R.A. Stockey, 2013), and Doyleales (i.e., *Doylea tetrahedrasperma* R.A. Stockey et Rothwell, 2009; Rothwell and Stockey, 2016). The discovery of *Xadzigacalix quatsinoensis* further increases the diversity of gymnosperms within the Apple Bay assemblage, emphasizing that North American biomes were systematically diverse immediately preceding the appearance of flowering plants in the fossil record.

All of the Apple Bay gymnosperms display some degree of seed enclosure that presumably functioned in pollination biology, seed protection, and/or seed dispersal (e.g., Leslie et al., 2018). Most conifer seeds, including all currently described from the Apple Bay flora (Klymiuk and Stockey, 2012; Atkinson et al., 2014a, 2014b), are more or less surrounded by parts of the bract/scale complex, while seeds of Bennettitaleans either are immersed among closely packed interseminal scales, or are more or less fused to each other (Rothwell et al., 2009; Rothwell and Stockey, 2010). Among the gnetophytes (e.g., *Protoephedrites eamesii*; Rothwell and Stockey, 2009), seeds are subtended by a pair of leaf homologues termed bracteoles, which form an outer “integument” or “envelope” in all living species of the clade (e.g., Chamberlain, 1935; Bierhorst, 1971; Friis et al., 2011). The seeds of *Doylea* spp. are enclosed within compound cones and more or less surrounded by a “cupule” (Rothwell and Stockey, 2016).

The new seed plant *Xadzigacalix* exhibits a cupulate syndrome that differs substantially from other enclosing syndromes of Apple Bay gymnosperms. Diagnostic characters for inferring organographic homology for “cupulate” gymnosperms include radial or bilateral symmetry of organ morphology; radial or bilateral anatomy of the cupulate organ; presence or absence of a bract subtending the cupulate organ; and presence or absence of the diagnostic seed plant leaf/branch trace vascular anatomy (Rothwell, 1976) of the cupulate organ. In contrast to all other gymnosperms at Apple Bay, the seeds of *Xadzigacalix* are orthotropous and radial, nonvascularized distal to the chalaza, and enclosed by a uni‐ovulate radial cupule of equivocal (but probably foliar) homologies. The uni‐ovulate cupule occurs at the apex of an organ with morphological and vascular characters like those of a stem (i.e., radial organ with a radial stele; Figures 11–13, 31, 8–10, 30). This novel combination of characters distinguishes *Xadzigacalix* not only from other Apple Bay seed plants, but all other gymnosperms.

### Insights from, and limits of, phylogenetic analyses

While both the sampling and taxonomic breadth of genome‐scale assessment of relationships among living plants is increasing rapidly (Ran et al., 2018b; Wan et al., 2018; Leebens‐Mack et al., 2019), models postulating or testing divergence hypotheses clearly indicate that major clades of living seed plants are evolutionarily separated from one another by hundreds of millions of years (Clark and Donoghue, 2017; Ran et al., 2018a). In most cases, “sister taxa” of living clades of non‐angiospermous seed plants are extinct and may as yet be entirely unknown from the fossil record (Rothwell et al., 2018). Phylogenetic hypotheses for seed plants thus remain equivocal owing to the reality that (1) systematic patterns resolved using only living plants frequently are altered when extinct terminals are added (e.g., Gauthier et al., 1988; Rothwell and Nixon, 2006; see Donoghue et al., 1989 for an analysis of this issue); (2) character sets are based on morphology, requiring nuance in both interpretation and methods of analysis (Coiro et al., 2018; Bateman 2020); (3) the “data set” of all available extinct taxa is incomplete; and (4) we currently have no reasonable method to estimate the severity and scale of these problems.

To develop phylogenetic hypotheses for the affinities of *Xadzigacalix*, we performed maximum parsimony cladistic analyses of a revised morphological matrix (Appendices S1, S2) for seed plants. The semi‐strict consensus of 60 most parsimonious trees tentatively suggests (BS < 50) that *Xadzigacalix* represents a lineage more closely allied with gnetophytes than other recognized gymnospermous plants (Figure 1–7, 21). Although the uni‐ovulate cupules of *Xadzigacalix* are superficially similar to those of some gnetophytes in terms of gross morphology, there are numerous organographic, vascular, and histologic differences that clearly distinguish the new plants from living and fossil Gnetales.

**Figures 22–29:**
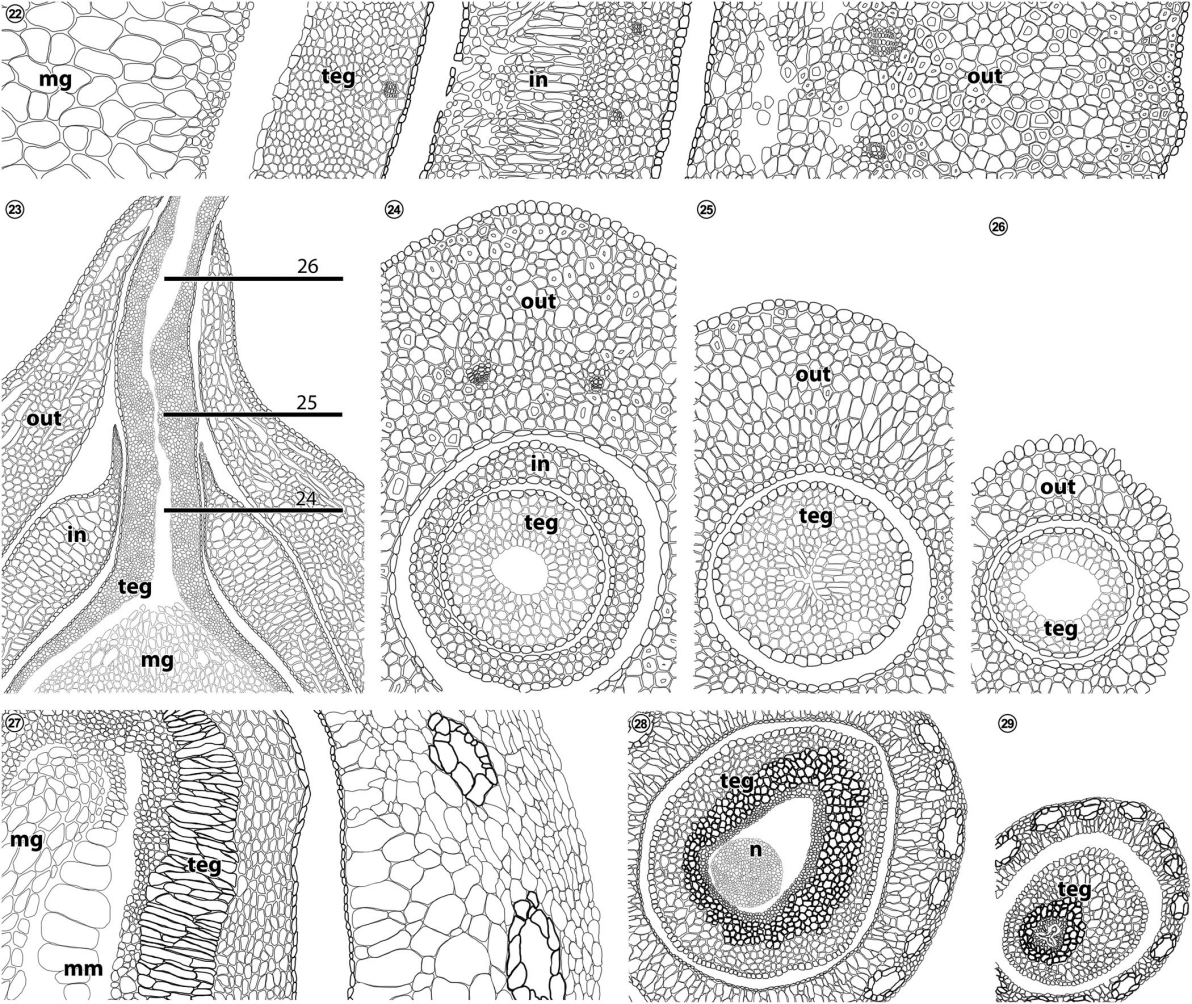
Comparison of integuments and seed‐enclosing structures of *Gnetum gnemon* with *Xadzigacalix quatsinoensis* gen. et sp. nov. **22**. Histology of *G. gnemon* in transverse section showing megagametophyte (mg), integument (teg), inner bracteole/“envelope” (in), and outer bracteole/”envelope” (out), illustrated from fig. 3 of Rodin and Kapil (1969). **23**. Longitudinal sections of *G. gnemon* developing ovule with enclosing inner (in) and outer (out) bracteoles with respect to integument (teg) and megagametophyte (mg), illustrated after figs. 6G, H of Takaso and Bouman (1986). Lines 24, 25, and 26 correspond to Figures 24–26, respectively. **24–26**. Transverse sections of the micropylar region of *G. gnemon* ovulate structures, showing the conformation of inner (in) and outer (out) bracteoles with respect to integumentary (teg) tissue of the micropyle. **27**. Histology of *X. quatsinoensis* in transverse section showing probable megagametophyte (mg), megaspore membrane (mm), integument (teg) comprised of endotesta, sclerotesta, and sarcotesta c.f. Figs. 5, 6, and surrounding cupule c.f. Figs. 3, 5. **28, 29**. Transverse sections of the micropylar region of *X. quatsinoensis*, comparable to Figures 24 and 25 (the distalmost portion of the micropyle is abraded in the fossil and thus unavailable for comparison); idealized illustrations after Figures 9, 10, and Appendix S3. Note presence of nucellus (n) within micropyle.

The seed‐bearing structures of gnetophytes have been a source of perplexity and debate for a considerable time (Thompson, 1916; Eames, 1952; Rodin and Kapil, 1969; Rothwell and Stockey, 2013). In most living and fossil gnetophytes, ovules are commonly surrounded by at least two layers of tissue (three layers, i.e., with the integument, for some species of *Gnetum*; Figures 22–26), which have been dubbed the “inner” and “outer envelopes”, or sometimes even referred to as “integuments”. Takaso and Bouman's study (1986) of the development of these structures in *Gnetum* L. have helped to clarify their organography. The “outer envelope” originates as a pair of opposite protuberances on either side of the ovule primordium (fig. 1I, 1J of Takaso and Bouman, 1986), fusing to enclose the developing ovule. The integument of the ovule elongates apically to form a micropyle that extends up to (and eventually out of) these outer bracteoles (fig. 3K, 3L of Takaso and Bouman, 1986). Meanwhile, a second pair of bracteoles initiates between the integument and outer whorl (fig. 3I, 3J of Takaso and Bouman, 1986), becoming the so‐called “inner envelope”. The complex, fleshy, enclosing structure surrounding most gnetalean seeds is therefore composed of opposite‐decussate (or whorled) bracteoles that may completely fuse as in *Gnetum*, or appear slightly separated as in *Ephedra* L. The micropyle‐forming tissue is of course the integument, which shows little to no histological differentiation into sarcotesta, sclerotesta, or endotesta (Rodin and Kapil, 1969).

At maturity, the integument of *Xadzigacalix* seeds is differentiated into sarcotesta, sclerotesta, and endotesta. These integumentary layers are superficially similar to the histology of the inner bracteole whorl of *Gnetum gnemon* L., as figured by Rodin and Kapil (1969). Some of these similarities may owe to specimen preparation and photography of *Gnetum* spp. (Rodin and Kapil, 1969). For instance, in Rodin and Kapil's (1969) micrographs, the slightly disorganized mesophyll‐like tissue of the “inner envelope” of *G. gnemon* appears darkened, heightening the resemblance to sclerotesta of *Xadzigacalix*, whereas sclereids in *G. gnemon* are represented as seemingly open voids and cells frequently indistinguishable from parenchyma. Nevertheless, the integument *Xadzigacalix* can be readily distinguished from the “inner envelope” of gnetaleans on the basis of distal regions of the micropyle, the tissue of which is consistent with the histological complexity of the seed (Figures 8–10, 22–29; Appendix S3).

It is also useful to examine the micropylar regions of these plants to illustrate that no “inner envelope” exists in *Xadzigacalix*. In *Gnetum*, the inner bracteoles tightly clasp the base of the micropyle; transverse sections taken below the outer envelope (Figure 24; fig. 6D of Takaso and Bouman, 1986) show that the micropyle is surrounded by a ring of inner bracteole tissue (note epidermal cells on inner/abaxial and outer/adaxial surfaces, clearly demarcating this ring of tissue as separate from the micropyle and the tissue of the fused outer bracteoles). In comparable sections of *Xadzigacalix*, the micropyle is surrounded only by cupule tissue (Figures 8–10, 22–29; Appendix S3). Similarly, when viewed in longitudinal section (Figures 1, 7), it is clear that the cupule of *Xadzigacalix* contains only the ovule. Apically, the tissues of gnetalean inner bracteoles are inflated into a void‐filling “flange” (Figure 23; fig. 6G–I of Takaso and Bouman, 1986). No such structures are evident in *Xadzigacalix* (Figure 1); the absence of similar tissues in *Xadzigacalix* is unlikely to be an artefact of preservation as even charcoalified gnetalean fossils show evidence of this tissue (fig. 2C of Friis et al., 2007). While the cupule of *Xadzigacalix* may conceivably have formed from fusion of bracteole‐like initials, there is no evidence for an inner whorl of tissue comparable to that seen in *Gnetum* spp. (Figures 1–7, 22–29; figs. 3–5 of Rodin and Kapil, 1969).

The cupule of *Xadzigacalix* is also distinct in that it contains large secretory ducts or canals, arranged in a ring around the entire periphery (Figures 1–7, 21, 1–7, 21). Although secretory structures in the form of laticifers do occur in extant gnetophytes, they are not present in the seed integument or in tissues surrounding the seed (Takaso and Bouman, 1986). Such laticifers are evident in mesophyll of juvenile *Gnetum* leaves and in stem tissues, becoming inconspicuous at maturity (Carlquist, 1996; Tomlinson and Fisher, 2005). Fossilized gnetalean leaves also have chemical signatures consistent with the presence of laticifers (Roberts et al., 2020). Unlike the conspicuous resin‐ or mucilage‐producing canals in the *Xadzigacalix* cupule (Figures 1–7, 21, 1–7, 21), however, gnetalean laticifers show little organization within leaf tissue, and are rarely larger than surrounding parenchyma cells (c.f. fig. 8 of Tomlinson and Fisher, 2005). Gnetalean laticifers branch or ramify (Carlquist, 1996), which is not a feature of the secretory structures of *Xadzigacalix* (Figures 14, 17). Moreover, the bracteoles surrounding gnetalean ovules do not contain obvious laticifers comparable to the secretory structures of the *Xadzigacalix* cupule; large void‐like structures in *G. montanum* Markgr. as figured by Rodin and Kapil (1969) are identified by these authors as sclereids.

The abundance and distribution of vascular tissues differs substantially between gnetophytes and *Xadzigacalix*. Vascular traces can be found in every tissue of the ovulate structure of *Gnetum*, including the seed integuments (Figure 22; Rodin and Kapil, 1969). In *Xadzigacalix*, by contrast, the cupule is unvascularized, as is the ovule (except at the chalaza). In this extinct species, three terete vascular strands diverge from the radial stele at the base of the cupule, and pass up to each vertex of the chalaza of the seed. The stele terminates as transfusion tissue below the nucellus (Figures 11–13, 31). By contrast, vascular tissues in the ovulate structures of *Gnetum* form two distinct rings of numerous vascular bundles at the level of the chalaza (see fig. 13 of Rodin and Kapil, 1969).

Another way in which *Xadzigacalix* fundamentally differs from gnetophytes is with respect to the nucellus: Gnetophytes have their nucellus fused to the seed integument for more than half the length of the seed and exhibit a pollen chamber (Chamberlain, 1935; Rothwell et al., 2009). By contrast, the nucellus of *Xadzigacalix* is free from the integument except at the chalaza (Figures 1–7, 21) and shows no evidence of a pollen chamber (c.f. fig. 27 of Rothwell et al., 2009); these nucellar characters are shared by *Xadzigacalix* and bennettitaleans (Rothwell et al., 2009). It is also worth noting that the uni‐ovulate cupule of the *Xadzigacalix* plant is borne terminally upon the homologue of a woody stem (Figure 20), whereas ovulate reproductive units of gnetophytes are borne in highly condensed cones or bisporangiate strobili (Ickert‐Bond and Renner, 2016). Finally, the vast majority of gnetophytes currently known from the Cretaceous fossil record have seeds that are four‐lobed or quadrangular in section (e.g., Friis et al., 2007, 2009, 2013, 2019a; Mendes et al., 2012, 2020); *Xadzigacalix*, by contrast, has a tetrahedral seed that is triangular in transverse section.

Although *Xadzigacalix* is possibly more closely related to gnetophytes than to any other gymnosperm lineage, it probably does not represent a true sister taxon. It is more likely that gymnosperm diversity remains underrepresented and that seed plant systematics are less fully resolved than commonly thought, owing to a paucity of anatomically preserved taxa throughout the Mesozoic. Phylogenetic placement of fossils using maximum parsimony cladistic analyses is also strongly hampered by probable homoplasy in morphological data sets. As an example, the analysis in this study resolved doylealean taxa within the coniferophytes, as opposed to sharing a sister taxon relationship with gnetophytes and Petriellales + flowering plants (Rothwell and Stockey, 2016). Placement of doylealean taxa within coniferophytes also distinguishes our results from those of Shi et al. (2021), whose analyses of a separate morphological matrix suggest that doylealean taxa share an affinity with Petriellales and Umkomasiales. We suspect that many differences between these analyses derive from character scoring interpretations of cupules either as wholly homologous organs (Shi et al., 2021) or as products of convergent evolution (this paper). Accurately ascertaining the affinities of various cupulate gymnosperms will require untangling the extent to which any of these structures are genuinely homologous.

### Morphology, anatomy, and homologies among seed‐bearing structures of “cupulate” Mesozoic gymnosperms

Mesozoic gymnospermous plants bearing seeds within a “cupule” are commonly referred to as “pteridosperms” or “Mesozoic seed ferns”, terms that reflect the traditional recognition that leaf and seed‐bearing structures of these plants are at least superficially similar to those of Paleozoic pteridosperms (Taylor et al., 2006, 2009a). While there is some emerging evidence for phylogenetic continuity for some of these plants across the Permian‐Triassic boundary (Kerp et al, 2001; Hamad et al., 2017; Blomenkemper et al., 2018, 2020; Anderson et al., 2019), most groups are unlikely to share close affinities with Paleozoic seed plants (Figure 1–7, 21). Instead, as is evident from lagerstätte like Apple Bay, many lineages of Mesozoic gymnosperms “experimented” with seed‐enclosing structures. As documented in our current investigation and in previous studies and reviews (e.g., Rothwell and Serbet, 1994; Taylor et al., 1994, 2006, 2009a), the cupules of most Mesozoic gymnosperm clades are not homologous organs (Figure 8–10, 30).

**Figure 30:**
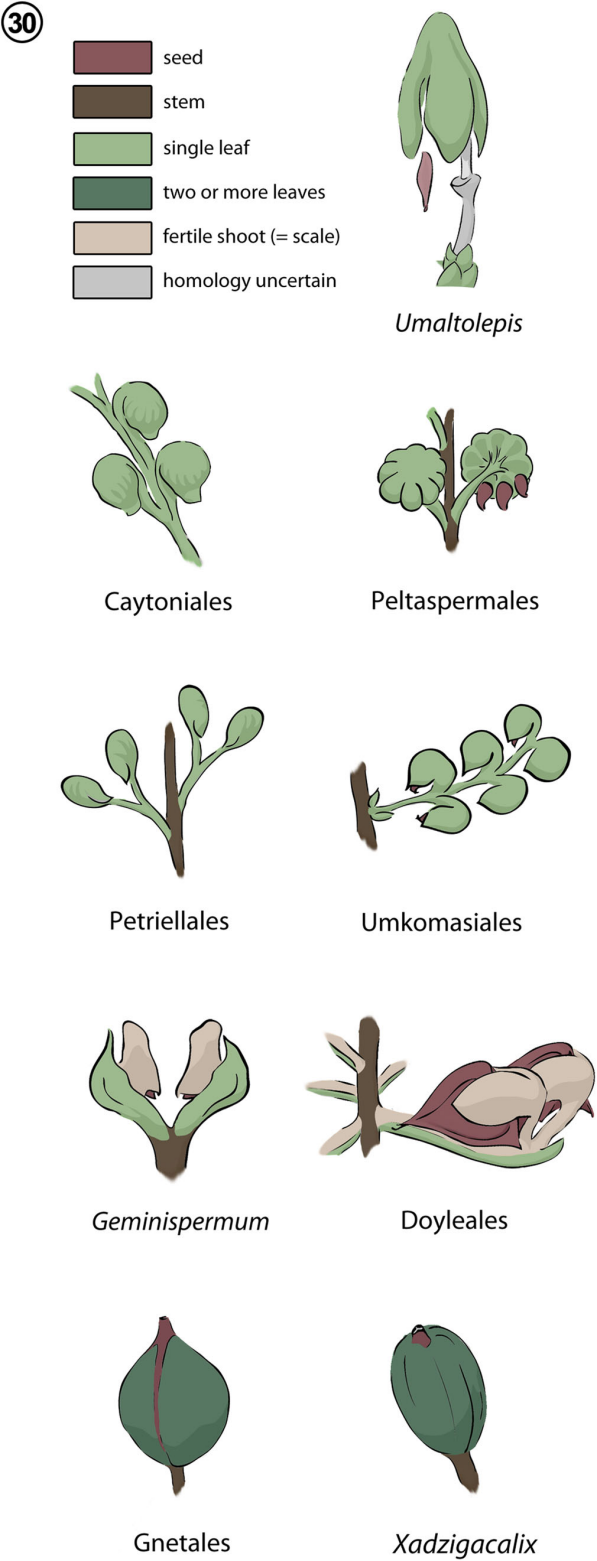
“Cupulate” Mesozoic gymnosperms. Structures are colored to indicate organographic homologues (key at upper left).

To clarify structural homologies among the heterogeneous assemblage of seed‐enclosing organs that characterize Mesozoic “cupulate” gymnosperms, conifers, and gnetophytes (including dispersed uni‐ovulate structures recognized by some authors as Erdtmanithecales; see Friis et al., 2009, 2013, 2019a; Mendes et al., 2020), we introduce and evaluate major groups of “cupulate” Mesozoic seed plants in terms of homologies to stem/leaf organography (Harris, 1976) and vascularization (Rothwell, 1976; Beck et al., 1982) intrinsic to each. This approach was originally advocated by Florin (1950) for conifers and later elaborated by Harris (1976) for gymnosperms in general and has previously been of great utility in revealing that the seed cones of conifers and taxads are derived from a more or less compound shoot system (e.g., Florin, 1951; Rothwell et al., 2011; Leslie et al., 2018). A comprehensive treatment of structural homologies for Mesozoic gymnosperms will be presented in a separate review. Here, we summarize syndromes for seed enclosure of “cupulate” Mesozoic gymnosperms for which anatomical data are available (Table 1, Figure 8–10, 30), and evaluate the organography of cupulate organs below.

**Table 1:** Features and developmental homologies of cupulate Mesozoic gymnosperms.

Fossil cupulate gymnosperms		Cupule construction	Cupule stalk in axil of leaf/bract	Ovules per cupule	Ovule shape	Apex of micropylar canal	Nucellar attachment	Sclerotic integument	Secretory structures	Age	Key references
Caytoniales	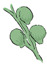	Abaxially inrolled lamina	–	multiple	ellipsoidal	simple	?	–		Jurassic	Harris, 1940, 1951, 1964; Reymanowna, 1973
Peltaspermales	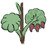	peltate stalk	–	multiple	ellipsoidal	simple	?	–		Triassic	Kerp, 1988; Poort and Kerp, 1990; Bomfleur et al., 2011
Petriellales	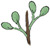	Ad?axially inrolled lamina	–	5‐6	ellipsoidal, triangular in xs	simple	at chalaza only	–	–	Triassic	Taylor et al., 1994
Umkomasiales	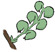	Abaxially inrolled lamina	–	1 (2)	ellipsoidal	bifid	at chalaza only	–?	+	Triassic	Klavins et al., 2002; Anderson et al., 2019
Doyleales	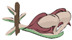	inverted stalk + 2 lateral lobes	yes	1	tetrahedral, triangular in xs	pollen horns	at chalaza only	+	–	Early Cretaceous	Stockey and Rothwell, 2009; Rothwell and Stockey, 2016; Shi et al., 2016, 2021
*Geminispermum*	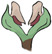	Inverted hood + stalk	yes	1	ellipsoidal, triangular in xs	?bifid	at chalaza only	–?	+?	Early Cretaceous	Friis et al., 2019a
Gnetales	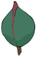	opposite‐decussate bracteoles	n/a	1	quadrangular	?bifid	chalaza to midway along seed body	+	+	Cretaceous	Friis et al., 2009, 2013 Rothwell and Stockey, 2013
*Umaltolepis*	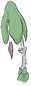	apically borne inverted lobes	?	4?	elongate, winged	?	?	–?	–?	Early Cretaceous	Herrera et al., 2017; Dong et al., 2019
*Xadzigacalix*	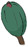	aril‐like?	n/a	1	tetrahedral, triangular in xs	triradiate opening	at chalaza only	+	+	Early Cretaceous	

*Note*: xs, cross section

**Figure 31:**
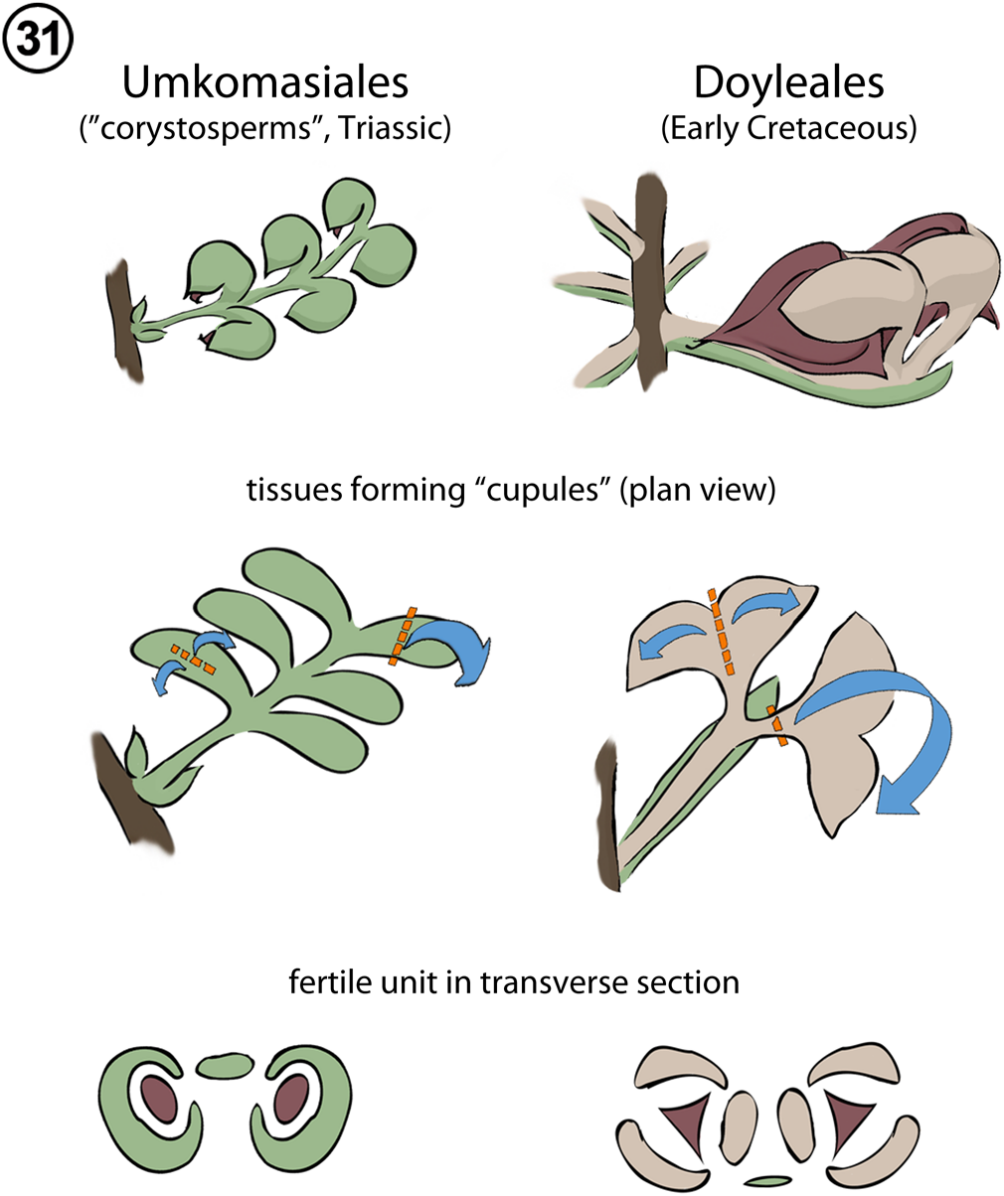
Derivation of cupules in umkomasialean and doylealean plants. Dashed lines and arrows (middle row) indicate planes and direction of folding/enrolling to accomplish enclosure of ovule (in cross section, lower row). Structures colored to indicate organographic homologues, as per Figure 8–10, 30.

Broadly, cupulate gymnosperms of the Triassic and Jurassic bore seeds upon foliar organs (=leaf homologues) with enrolled and/or recurved laminar tips forming the “cupules” (Figures 8–10, 30, 11–13, 31); these include Caytoniales, Peltaspermales, Umkomasiales, and Petriellales. Where such leaf homologues are known to be borne on a stem homologue, they form a simple fertile shoot system (sensu Anderson and Anderson, 2003; Anderson et al., 2019). By contrast, “cupulate” gymnosperms that appear first in the Cretaceous bear seeds and their enclosing “cupule” either apically or laterally upon a stem‐like structure (*Umaltolepis mongoliensis* Herrera et al., 2017; Dong et al., 2019; Nosova, 2020) and/or they bear seeds on compound shoot systems (e.g., Gnetales; Ickert‐Bond and Renner, 2016). For some taxa (i.e., *Doyleales*, *Geminispermum virginiense*), these compound shoot systems are broadly comparable to those of extant conifer seed cones, in that they are composed of two orders of cauline and foliar organs (Figure 11–13, 31).

***Caytoniales***: Seed‐bearing cupules of Caytoniales are multiovulate and occur apically on unbranched lateral units that appear to be pinnately arranged (e.g., Thomas, 1925). The cupule is formed of a bilaterally expanded distal region that is abaxially inverted and laterally enrolled to form a cupule wall with a basally directed lip through which pollination occurs (Harris, 1951, 1964; Reymanowna, 1973; Dilcher, 1979). These cupulate units are parts of a pinnate organ, in which their bilateral morphology indicates that they represent foliar homologues. This interpretation is consistent with the absence of a bract subtending each unit, but remains to be confirmed by the discovery of permineralized specimens with preserved vascular anatomy.

***Peltaspermales***: “Cupules” of peltasperms are either laminar (i.e., *Autunia conferta* J.H.F. Kerp, 1988) or peltate, have several abaxially borne seeds, and are helically arranged on an axis (Kerp, 1988; Poort and Kerp, 1990; Kerp et al., 2001). This morphology and the absence of a subtending bract imply that individual cupulate units are foliar and part of a simple shoot. As with Caytoniales, the morphological interpretation of homology for peltasperm cupulate systems remains to be confirmed by the discovery of vascular anatomy.

***Umkomasiales***: The organography of “cupulate” fructifications of umkomasialean plants has been subject to significant debate (Shi et al., 2016, 2019; Rothwell and Stockey, 2016; Anderson et al., 2019). Here, we clarify that umkomasialean plants produce cupulate seeds upon structures that are homologous to leaves. Umkomasialean plants produce lateral cupule‐bearing units of varying complexities, borne in a helical arrangement upon a larger axis (e.g., Anderson et al., 2019). Each lateral unit (Figure 11–13, 31) typically bears paired uni‐ovulate (rarely bi‐ovulate, see Klavins et al., 2002) cupules (Thomas, 1933; Holmes and Ash, 1979; Holmes, 1987; Klavins et al., 2002; Anderson and Anderson, 2003; Pattemore, 2016; Anderson et al., 2019). Species with simple lateral units may bear only a single cupule (e.g., *Umkomasia resinosa* S. Klavins, T.N. Taylor & E.L. Taylor, 2002), while other species have two or more terminal cupules (ranging up to ~14 in *U. dissecta* J.M. Anderson and H.M. Anderson, 2003; Anderson et al., 2019), that are more or less arranged in opposite pairs. Where lateral units contain multiple pairs of cupules, they are occasionally subtended by additional spine‐like appendages termed “bracteoles” by Thomas (1933) and Anderson et al. (2019). Where one scale‐like appendage is present at the base of a cupulate unit, that structure has been interpreted by Thomas (1933) and several more recent authors (e.g., Anderson and Anderson, 2003) as a subtending bract. However, there are numerous specimens that show no evidence of a subtending bract (e.g., plate 85, fig. 1 and Plate 87, figs. 8 and 9 of Anderson and Anderson, 2003; figs. 1, 2, and 4 of Shuqin et al., 2008). It is the presence or absence of a subtending bract that is at the heart of opposing interpretations for the homologies of the lateral cupulate units of *Umkomasia* spp. (Rothwell and Stockey, 2016; Shi et al., 2016, 2019). If each unit is subtended by a bract (=leaf homologue), then the cupulate unit must represent an axillary shoot (Harris, 1976), and the base of the cupulate units will have radial vascular architecture, as is characteristic of stems. If, on the other hand, the cupule‐bearing lateral units of *Umkomasia* spp. are not subtended by a bract, are not radial, and have bilateral vascular anatomy, then they represent leaf homologues as interpreted by Rothwell and Stockey (2016) and Anderson et al. (2019).

Anatomically preserved cupulate systems of *Umkomasia resinosa* provide data needed to resolve these competing hypotheses (Klavins et al., 2002). Ovulate fructifications of *U. resinosa* consist of a radial axis with a radial stele (diagnostic of a stem homologue) that produces bilateral cupulate laterals with bilateral vascular anatomy (diagnostic of a leaf homologue). Moreover, there clearly is no bract or bract trace subtending each of the cupulate units, thus strongly supporting the hypothesis that cupulate units of umkomasialean plants are leaf homologues borne on a stem homologue (Figure 11–13, 31). The cupule wall in *Umkomasia* spp. therefore consists of a broadened laminar (i.e., leaf or leaflet homologue) tip that is inverted and inrolled toward the abaxial surface, producing a cupule that consists entirely of leaf tissue (Klavins et al., 2002; Anderson et al., 2019; Figure 11–13, 31).

***Petriellales***: ‘Cupules’ of Petriellales are formed from a bilaterally expanded distal region of a leaf or leaflet homologue that is inverted and laterally enrolled to enclose several seeds (Taylor et al., 1994). As originally interpreted, cupulate axes of *Petriellaea triangulata* T.N Taylor, G.M. del Fueyo & E.L. Taylor occur in pairs at the apex of a forking structure that may be borne on a subtending axis in a lax simple strobilus (Bomfleur et al., 2014). However, this interpretation has yet to be verified by the discovery of articulated cupulate systems (B. Bomfleur, Westfälische Wilhelms‐Universität Münster, personal communication, 2021). Nevertheless, both the bilateral morphology and vascular anatomy of the *P. triangulata* cupulate organ are consistent with the interpretation that such cupules represent foliar homologues (Klavins et al., 2002). As in *Caytonia* Harris and *Umkomasia* Thomas, the wall of *Petriellaea* cupules is formed from a laterally expanded, laminar pinnule or pinna tip. In contrast to all other known Mesozoic gymnosperm cupules, those of *Petriellaea* have been described as being inrolled with the adaxial surface of the cupule wall positioned to the inside (rather than to the outside) and have adaxially borne seeds (Taylor et al., 1994). The adaxial seed attachment for *Petriellaea* has been questioned recently (Shi et al., 2021), and more evidence is necessary to resolve the question. The nature of seed attachment is a most important character because *Petriellaea* is the only seed plant other than angiosperms reported to bear seeds on the adaxial surface of the enclosing structure.

***Gnetales***: Among living species of the Gnetales, seeds occur at the tip of the axis (i.e., a stem homologue; Harris, 1976; Figure 8–10, 30) within a compound cone (Chamberlain, 1935; Sporne, 1965; Bierhorst, 1971). Based on the transformational series of morphologies proposed by Eames (1952) and fossil evidence for an extinct intermediate in that series (Rothwell and Stockey, 2013), the cauline attachment of gnetophyte seeds appears to be the result of reduction and condensing from an ancestral condition where seeds are borne on fertile shoots in the axils of leaves (see fig. 8 of Rothwell and Stockey, 2013). This condition is comparable to that in the conifer *Taxus* L., in which seeds are terminal (i.e., on a stem homologue) in the axil of a leaf on an otherwise vegetative leafy shoot system, but may actually represent a highly condensed and reduced compound cone (e.g., Leslie et al., 2018).

***Doyleales***: The ovulate reproductive structures of a number of “cupulate” Mesozoic gymnosperms can be equated to the compound seed cones of conifers, wherein the seed‐bearing and seed‐enclosing structure is attached to a cone axis and subtended by a bract (Figure 11–13, 31). Such homologies are most obvious for species of the Doyleales (i.e., *Doylea tetrahedrasperma*, *D. mongolica*, an unnamed new doylealean species, Shi et al., 2021; Figures 8–10, 30, 11–13, 31), and for dispersed bract/scale complexes of two additional species originally described as *Umkomasia* (i.e., *U. corniculata* and *U. trilobata* G. Shi, P.R. Crane, Herend., Ichinorov, Mas.Takah, & F. Herrerra, 2019), but clearly assignable to *Doylea* on the basis of compound construction. The bract/scale complexes of all of these species divide at the base to produce a bract abaxially and the cupulate unit adaxially. The cupulate unit then divides laterally to produce one (unnamed new species; Shi et al., 2021), two (*D. tetrahedrasperma*; Stockey and Rothwell, 2009), or three (*D. trilobata*; Shi et al., 2019) ovuliferous scale homologues, each of which terminate in a uni‐ovulate cupule bearing a tetrahedral seed (Figures 8–10, 30, 11–13, 31; c.f. Rothwell and Stockey, 2016 to Shi et al., 2019). The recently described, unnamed doylealean from Upper Mongolia (Shi et al., 2021) illustrates a condition in which the cupulate unit does not undergo a lateral division to form two fertile stalks (forked ovuliferous scale homologue). Instead, the two vascular strands persist within a single stalk, bifurcating to vascularize two seeds within a bi‐ovulate cupule (see extended data fig. 3 of Shi et al., 2021). Doylealean cupules are formed by lateral inflation of the distal (inverted or recurved) portions of fertile stalk/ovuliferous scale (Figure 11–13, 31). Thus, when viewed in cross section, doylealean cupules consist of fertile stalk tissue on one side of the seed and laminar lobes along the other sides of each seed (c.f. figs. 24–26 of Rothwell and Stockey, 2016, fig. 4 of Shi et al., 2016, fig. 6 of Shi et al., 2019).

***Geminispermum virginiense***: Less‐obvious compound seed cone structure and homologies are displayed by *G. virginiense* (Friis et al., 2019b), which constitutes an axis that branches to form two units, each consisting of an abaxial bract and an adaxial uni‐ovulate cupule (Figure 8–10, 30; Friis et al., 2019b). Because the base of this fossil is radial and has a radial vascular system (fig. 2E of Friis et al., 2019b), it is comparable to a stem in both morphology and vascular anatomy. Distal to the branching of the primary axis or main stem, each cupulate unit has a C‐shaped vascular strand (fig. 2D of Friis et al., 2019b), which branches into three traces; the central trace enters the bract, and the two lateral traces enter the base of the cupulate structure (fig. 3 of Friis et al., 2019b). This vascular syndrome is strictly comparable to the vascularization of a leaf and accompanying axillary branch on the vegetative system of typical gymnosperms (Rothwell, 1976). Thus, we interpret *G. virginiense* to be a determinate compound seed cone composed of a single pair of oppositely arranged bract/scale complexes. This morphology is slightly simpler in overall cone structure, but otherwise comparable to the oppositely arranged bract/scale seed cone complexes of some Cupressaceae [i.e., one opposite pair, rather than two decussate pairs; e.g., *Tetraclinis articulata* (Vahl) Mast., fig. 4a of Farjon, 2005] or the bract‐scale complexes of some living podocarps (e.g., Chamberlain, 1935; Sporne, 1965).

***Xadzigacalix quatsinoensis***: The uni‐ovulate cupule of *Xadzigacalix* is borne apically upon a radial stem homologue exhibiting a radial stele with secondary growth, i.e., wood. The stele ramifies within the base of the cupule to produce three terete strands that terminate at the base of the seed. There is no histological evidence that the cupule of *Xadzigacalix* is constructed of fused leaf homologues like the bracteoles of gnetophytes because the three vascular strands do not pass into the enclosing tissue of the cupule. Rather, it appears to be more like the aril of some taxaceous conifers (see Farjon, 2005). Additional specimens of this plant are needed to resolve whether the cupulate units are solitary upon the stem or borne in an aggregate (cone‐like) fructification.

## SUMMARY AND CONCLUSIONS

Relationships among disparate clades of Paleozoic and Mesozoic non‐coniferophyte gymnosperms remain incompletely resolved (Nixon et al., 1994; Hilton and Bateman, 2006; Taylor et al., 2006; Taylor and Taylor, 2009b; Rothwell and Stockey, 2016). This state of affairs owes largely to a combination of incomplete and differential preservation of the fossils, inconsistent terminology used for the structures of different taxa, and a lack of understanding for the underlying homologies of the various structures that make up the cupulate system. As detailed here, cupules and cupule‐bearing structures show a wide range of structural heterogeneity among the various groups of Mesozoic gymnosperms and almost certainly do not represent homologous organ systems across the known range of taxonomic groups (Figure 8–10, 30, Table 1). The hypotheses we have introduced here, following Harris (1976), provide a working framework to interpret new additions to the gymnosperm fossil record.

Several clades of cupulate Mesozoic gymnosperms have sparked interest as possible antecedent or sister clades to flowering plants (Harris, 1964; Doyle, 2006, 2014; Stockey and Rothwell, 2009; Taylor and Taylor, 2009a; Doyle and Endress, 2011). The apparent abrupt appearance of angiosperms in the fossil record (Herendeen et al., 2017; Bateman, 2020) and the persistent difficulty in ascertaining their phylogenetic context with respect to other spermatophytes has been called an “abominable mystery” (attributed to Darwin; e.g., Davies et al., 2004; but see Friedman, 2009; Buggs et al., 2021). It is becoming obvious that some of this mystery stems from a woefully incomplete sampling of outgroup taxa. Most extant gymnosperm species are relicts of clades that were more highly diverse throughout the Mesozoic. Until recently (i.e., Rothwell and Stockey, 2016; Herendeen et al., 2017), it was assumed that most major lineages of gymnospermous plants had been recognized in the fossil record. Their evolutionary relationships to one another might remain inscrutable, but few botanists seriously entertained the opinion that we might still be missing wide swathes of vascular plant diversity. The unprecedented organography exhibited by the *Xadzigacalix quatsinoensis* plant—which has a tetrahedral seed with complex integument within a resinous cupule, borne apically upon a woody stem—illustrates that we still stand to discover new clades of Mesozoic gymnosperms. *Xadzigacalix* likely constitutes a new order of seed plants, perhaps with affinities to gnetophytes or angiosperms. Given that we are still discovering novel gymnosperm clades from the geologic interval immediately antecedent to the rapid diversification of angiosperms, *Xadzigacalix* fossils also underscore that we continue to lack crucial fossil data necessary for understanding the evolutionary context of flowering plants.

## AUTHOR CONTRIBUTIONS

This study was conceived by A.A.K., G.W.R., and R.A.S. Specimens were discovered and prepared by A.A.K. and photographed by G.W.R. and A.A.K.; cladistic analyses and character scoring were performed by G.W.R., A.A.K., and R.A.S.; 3D reconstructions, photographic plates, illustrations, and cladograms were created by A.A.K. The manuscript was written and revised by A.A.K., G.W.R., and R.A.S.

## Supporting information


**Appendix S1**. Morphological matrix for cladistic analysis (NEXUS file format), including a tree block of 60 most‐parsimonious trees found under parsimony ratchet perturbation. Archived as Morphobank Project P4231 and also available as a Winclada‐executable file by request.

**Appendix S2**. Table of concordance explaining character revisions and character state changes made to the previous version of this matrix published by Rothwell and Stockey ( 2016; Morphobank Project 23745).

**Appendix S3**. Serial sections of apical/micropylar region and seed chalaza/cupule base of *Xadzigacalix quatsinoensis* in PowerPoint (.ppt) format.

## Data Availability

Our morphological matrix for seed plant phylogeny is available as Appendix S1 and archived as Morphobank Project P4231. Serial section “labelfield” image files for 3D reconstruction in Avizo/Amira are available via Figshare (https://doi.org/10.6084/m9.figshare.19597300, https://doi.org/10.6084/m9.figshare.19597453) or by request.
