# Supplementary material for: A novel cupulate seed plant, Xadzigacalix quatsinoensis gen. et sp. nov., provides new insight into the Mesozoic radiation of gymnosperms
Source: Am J Bot. 2022 Jun 14;109(6):966–85. doi: 10.1002/ajb2.1853 (PMC9328379; doi:10.1002/ajb2.1853)
Supplement: Supplementary file 2 — Appendix S2. Table of concordance explaining character revisions and character state changes made to the previous version of this matrix published by Rothwell and Stockey ( 2016; Morphobank Project 23745). [file AJB2-109-966-s002.pdf]

Appendix S2: Table of concordance explaining character revisions and character state changes made to the previous version of this matrix published by Rothwell and Stockey (2016; Morphobank Project 23745).

Character in Klymiuk et al. 2022	Corresponding character in Rothwell & Stockey 2016	Character changes in new matrix	*Taxon* (prev. score → new score)
[1] Maule reaction	[2]		
[2] Radicle	[5]	Re-scored	*Glossopteris* (– → 0)
[3] Veg. short shoots	[6]		*Umkomasia* (1 → 0)<br>*Caytonia* (1 → ?)<br>*Gnetum* (– → 0)
[4] Habit	[7]		
[5] Branching	[8]		
[6] Persistent leaf bases	[9]		
[7] Cataphylls (bud scales)	[11]		*Medullosa* (– → 0)
[8] Leaves_1	[10]		
[9] Vegetative leaves_1		New character, revision of [12,13]	
[10] Vegetative leaf dissection		New character, revision of [12, 13]	
[11] Vein orders		New character	
[12] Laminar venation	[17]	Charstate revision	*Medullosa* (0 → 2,3)<br>*Ginkgo* (0 → 1)<br>*Cordaixylon* (0 → 2)<br>*Mesoxylon* (0 → 2)<br>*Umkomasia* (0 → 1)<br>Peltaspermales (0 → 1)<br>*Glossopteris* (2 → 2,3)<br>*Caytonia* (2 → 3)<br>Cycadaceae (0 → 0,2)<br>Zamiaceae (0 → 0,2)<br>Bennettitales (0 → 2,3)<br>Pentoxylon (0 → 3)<br>*Ephedra* (– → 4)<br>*Welwitschia* (2 → 4)<br>*Gnetum* (2 → 4)

			angiosperms (1 → 4)<br>*Doylea tetrahedrasperma* (? → 3)
[13] Leaf trace divergence	[14]		*Umkomasia* (– → 1)<br>angiosperms (– → 1)
[14] Leaf traces	[15]		*Umkomasia* (0 → 1)
[15] Girdling leaf traces	[16]		
[16] Guard_cell_poles	[18]		
[17] Stomata	[19]		
[18] Foliar_sclereids	[20]		
[19] Secretory_structures	[22]	Charstate revision	*Elkinsia* (1 → 0)<br>*Heterangium* (1 → 0)<br>*Lyginopteris* (2 → 0)<br>*Callistophyton* (3 → 1)<br>*Quaestora* (3 → 0)<br>*Medullosa* (3 → 0)<br>*Emporia* (1 → 0)<br>*Pinus* (3 → 2)<br>*Podocarpus* (3 → 2)<br>*Taxus* (3 → 2)<br>*Cordaixylon* (2 → 3)<br>*Mesoxylon* (1 → 2)<br>*Umkomasia* (2 → 1)<br>Bennettitales (2 → 0)
[20] Apical meristem	[23]		
[21] Protoxylem_architecture	[24]		
[22] Polystele	[25]		
[23] Primary xylem	[26]		
[24] Metaxylem	[27]		
[25] Secondary xylem	[28]	Charstate clarification	Cycadaceae (– → 1)<br>Zamiaceae (– → 1)<br>Bennettitales (– → 1)<br>*Pentoxylon* (– → 1)<br>*Ephedra* (– → 1)<br>*Welwitschia* (– → 1)<br>*Gnetum* (– → 1)<br>angiosperms (– → 1)<br>*Doylea tetrahedrasperma* (– → 1)<br>*Doylea mongolica* (– → 1)

			Petriellales (– → 1)
[26] Tertiary_helical_thickenings_in_tracheids	[29]		*Ginkgo* (– → 0)
[27] Vessels	[31]		
[28] End_wall_pit_or_vessel_perforations	[30]		
[29] Rays	[32]		
[30] Companion cells in phloem	[33]		
[31] Sieve_tube_plastid_inclusions	[34]		
[32] Structures bearing megasporangia (fertile units)	[35]	Charstate revision	*Quaestora* (0 → ?)<br>*Ginkgo* (3 → 2)<br>*Taxus* (4 → 5)<br>*Glossopteris* (0 → 2)<br>*Caytonia* (0 → ?)<br>angiosperms (– → 5)<br>*Doylea mongolica* (? → 4)
[33] Shape of ovule_bearing_structure		New character	
[34] Microsporangium_bearing_structures	[36]	Charstate revision (new state5)	*Ginkgo* (3 → 2)<br>Peltaspermales (? → 0)<br>*Caytonia* (1 → ?)<br>angiosperms (– → 5)<br>Petriellales (3 → ?)
[35] Symmetry of megasporangium-bearing or ovuliferous fertile shoot	[37]		*Pentoxylon* (0 → 1)
[36] Complexes of ovuliferous_fertile_shoots	[38]		*Pentoxylon* (0 → 1)
[37] Ovuliferous_fertile_shoot_complexes	[39]		*Ginkgo* (1 → 0)
[38] Bract_and_axillary_ovulate_shoot_complexes	[40]		*Ginkgo* (1 → 0)
[39] Megasporangia_and_microsporangia	[41]		
[40] Megasporophylls, morphology	[42]	Charstate revision (major)	
[41] Microsporophylls, morphology	[43]	Charstate revision (major)	
[42] Microsporophylls2	[44]		*Cordaixylon* (0 → 1)<br>*Mesoxylon* (0 → 1)<br>*Caytonia* (0 → ?)<br>Bennettitales (– → 0)<br>*Pentoxylon* (1 → 0)<br>*Gnetum* (– → 1)<br>*Doylea tetrahedrasperma* (? → 0)<br>*Doylea mongolica* (? → 0)
[43] Microsporangia_per_sporophyll	[45]		*Ginkgo* (0,1 → 1)

[44] Stamen numbers	[46]		
[45] Microsporophylls3	[47]		
[46] Microsporophyhlls4	[48]		
[47]Stamen form	[49]		
[48] Position_of_microsporangia (CHECK state3?)	[50]		
[49] Microsporangia	[51]		
[50] Microsporangia_dehiscence	[52]		
[51] Inner_staminodes	[53]	Re-scored	*Ginkgo* (0 → –)
[52] Position_of_attachment_of_megasporangium or ovule upon leaf or shoot homologue	[56]		*Medullosa* (– → 0)
[53] Ovule/megasporangium_orientation	[57]		
[54] Integument1	[58]		Zamiaceae (5 → 2)
[55] Integumentary vascularization	[59]	Re-scored	*Ginkgo* (– → 2)<br>*Umkomasia* (– → 2)<br>*Glossopteris* (– → 2)<br>*Pentoxylon* (– → 2)<br>*Ephedra* (– → 2)<br>*Welwitschia* (– → 1)<br>*Gnetum* (– → 0)
[56] Histology of integument	[60]	Charstate revision	*Elkinsia* (– → 0)
[57] Sarcotesta	[64]		
[58] Anatomical_symmetry_of_ovule	[61]		*Pinus* (– → 1)<br>*Podocarpus* (– → 0)<br>*Taxus* (1 → 0)<br>*Ephedra* (– to 0)
[59] Fusion_of_integument_to_nucellus	[62]		Peltaspermales (0 → ?)<br>*Caytonia* (0 → ?)
[60] Tubular_micropyle	[63]	Charstate revision	Bennettitales (2 → 1)
[61] Integument_apex_sealing_post_pollination	[65]		
[62] Pollen chamber	[66]	Charstate clarification	
[63] Central column	[67]		
[64] Lagenostome/salpinx/nucellar_beak	[68]		
[65] Post-pollination_pollen_chamber_sealing	[69]		
[66] Megasporangium/nucellus_vascularization	[70]		
[67] Nucellar_cuticle	[71]		
[68] [pollen] Testa	[72]		
[69] [pollen] Exotesta	[73]		

[70] Ruminations_in_the_seed_coat	[74]		
[71] Enclosure of ovule or homologue	[75]	Charstate revision (+ states)	*Medullosa* (– → 0)<br>*Podocarpus* (2 → 5)<br>*Pentoxylon* (– → 0)<br>*Ephedra* (0 → 2)<br>*Welwitschia* (0 → 2)<br>*Gnetum* (0 → 4)
[72] Meio_or_megasporangium_or_ovule	[76]		
[73] Closed carpel with stigmatic pollen germination	[77]		
[74] Carpel_number	[78]	Re-scored	*Doylea tetrahedrasperma* (– → 6)<br>*Doylea mongolica* (– → 6)<br>Petriellales(– → 6)
[75] Hypanthium	[79]		
[76] Placentation	[80]		
[77] Carpels	[81]		
[78] Ovules per carpel	[82]		
[79] Perianth	[83]		
[80] Perianth_symmetry	[84]		
[81] Microspore/pollen_cytokinesis	[85]		
[82] Miospore/pollen germination area	[86]		*Ephedra* (2 → 1)<br>*Gnetum* (– → 0)
[83] Microspore/pollen_symmetry	[87]		
[84] Microspores/pollen	[88]		
[85] Infratectal_structure	[89]		
[86] Tectum	[90]		
[87] Exine striations	[91]		*Ephedra* (1 → 0)
[88] Supratectal_spinules	[92]		
[89] Aperture_membrane	[93]		*Ephedra* (– → 0)
[90] Endexine	[94]		
[91] Microgametophyte nuclei	[95]		*Ginkgo* (– → 0)<br>*Ephedra* (0 → 1)
[92] Sterile_cell	[96]		*Ephedra* (0 → –)
[93] Sperm	[97]		
[94] Megaspore_tetrad	[98]		*Welwitschia* (– → 1)<br>*Gnetum* (– → 1)
[95] Megaspore wall	[99]		
[96] Megagametophyte1	[100]		*Ephedra* (0 → 1)

[97] Megagametophyte2	[101]	*Ephedra* (0 → 1)
[98] Megagametophyte_cellularization	[102]	*Ephedra* (0 → 1)
[99] Sperm_transfer	[103]	
[100] Embryogenesis (fusion of sperm)	[104]	*Welwitschia* (– → 0)
[101] Nutritive_tissue_in_seed	[105]	
[102] Embryo	[106]	
[103] Proembryo	[107]	
[104] Secondary_suspensor_(embryonal_tubes)	[108]	
[105] Feeder_in_embryo	[109]	
[106] Postzygotic quiescence and/or seed dormancy	[110]	
[107] Seed_germination	[111]	
