# Supplementary material for: A novel cupulate seed plant, Xadzigacalix quatsinoensis gen. et sp. nov., provides new insight into the Mesozoic radiation of gymnosperms
Source: Am J Bot. 2022 Jun 14;109(6):966–85. doi: 10.1002/ajb2.1853 (PMC9328379; doi:10.1002/ajb2.1853)
Supplement: Supplementary file 3 — Appendix S3. Serial sections of apical/micropylar region and seed chalaza/cupule base of Xadzigacalix quatsinoensis in PowerPoint (.ppt) format. [file AJB2-109-966-s001.pptx]

## Slide 1
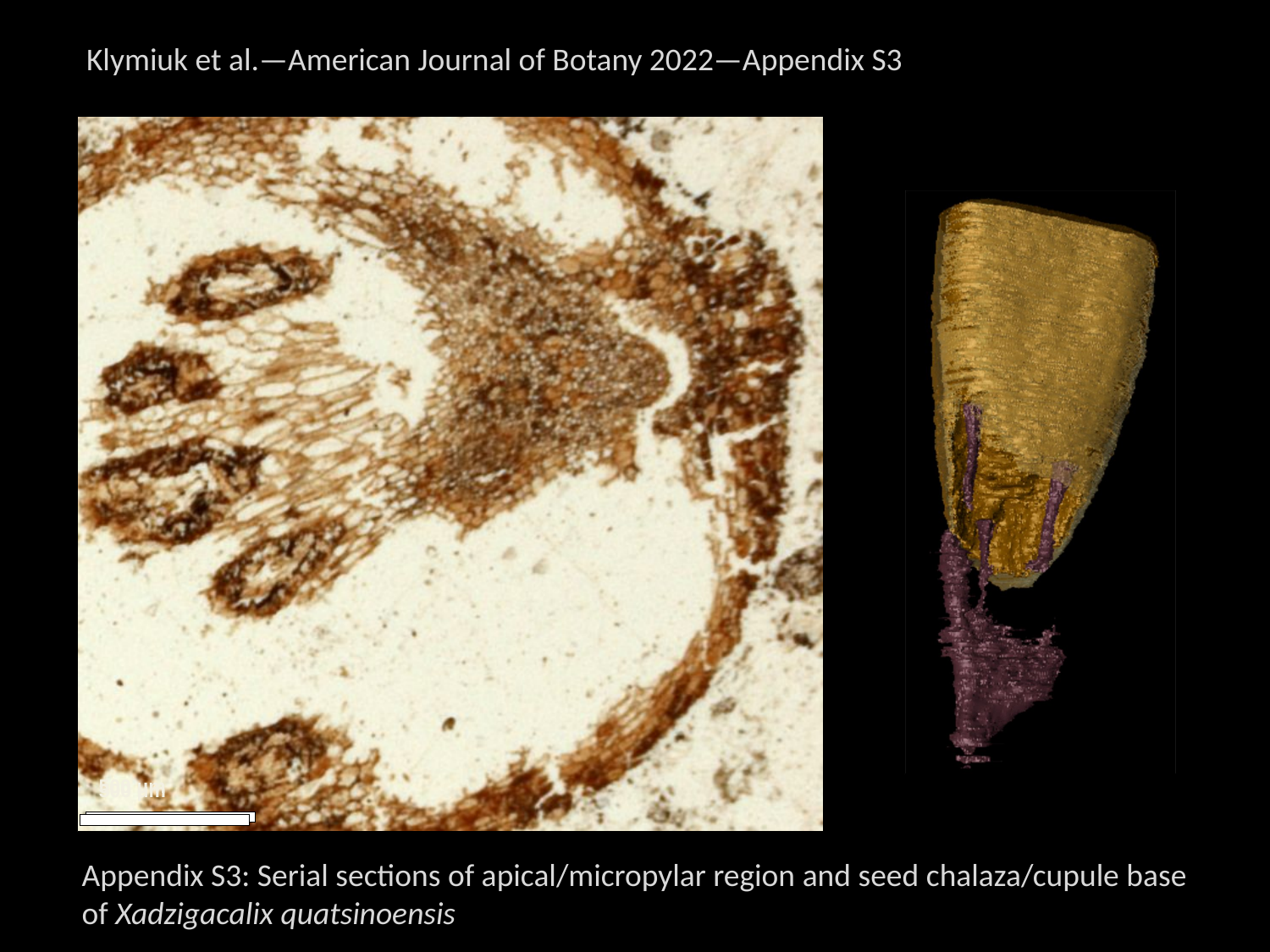

Klymiuk et al.—American Journal of Botany 2022—Appendix S3
500 μm
500 μm
Appendix S3: Serial sections of apical/micropylar region and seed chalaza/cupule base of Xadzigacalix quatsinoensis

## Slide 2
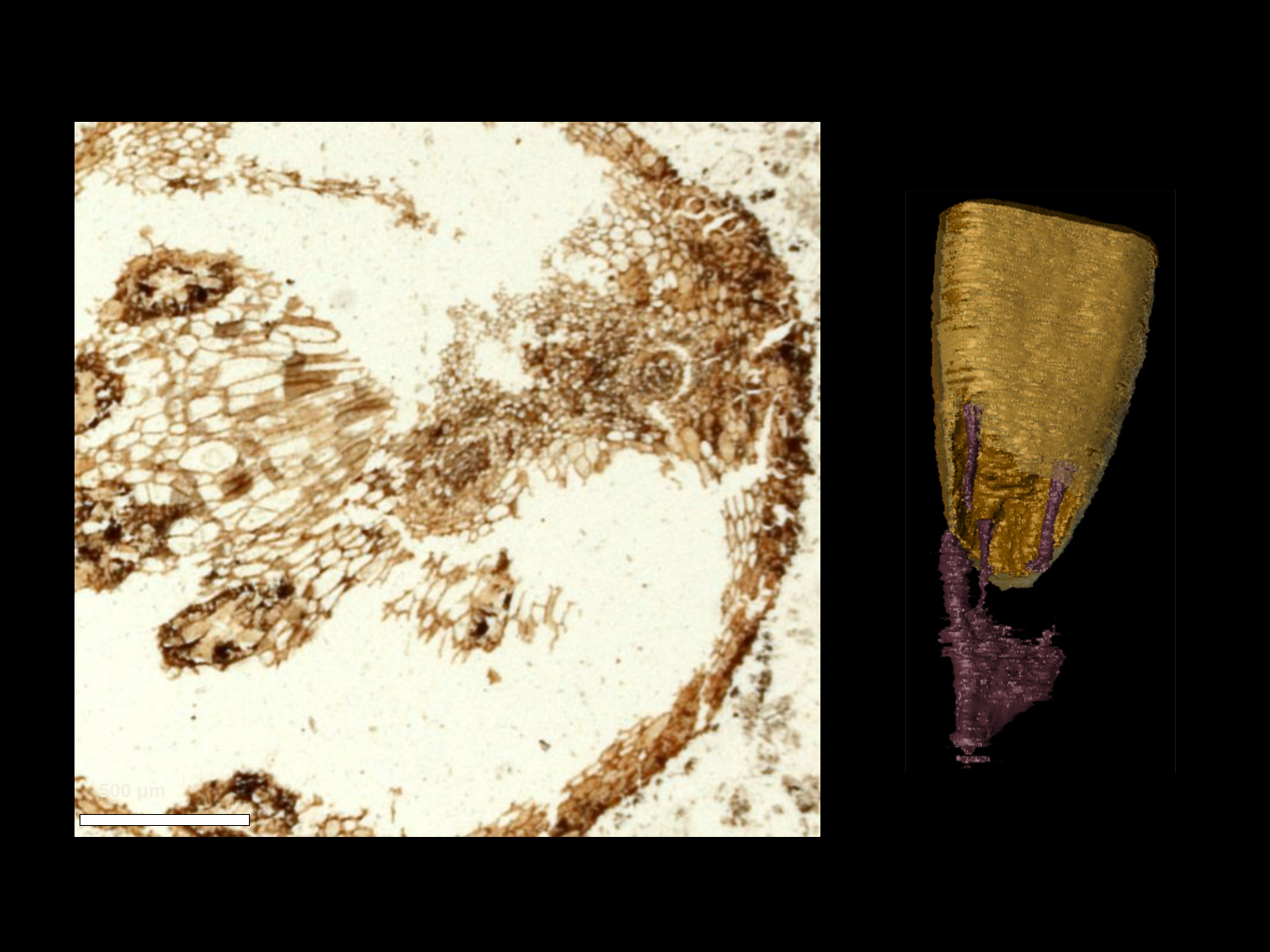

500 μm

## Slide 3
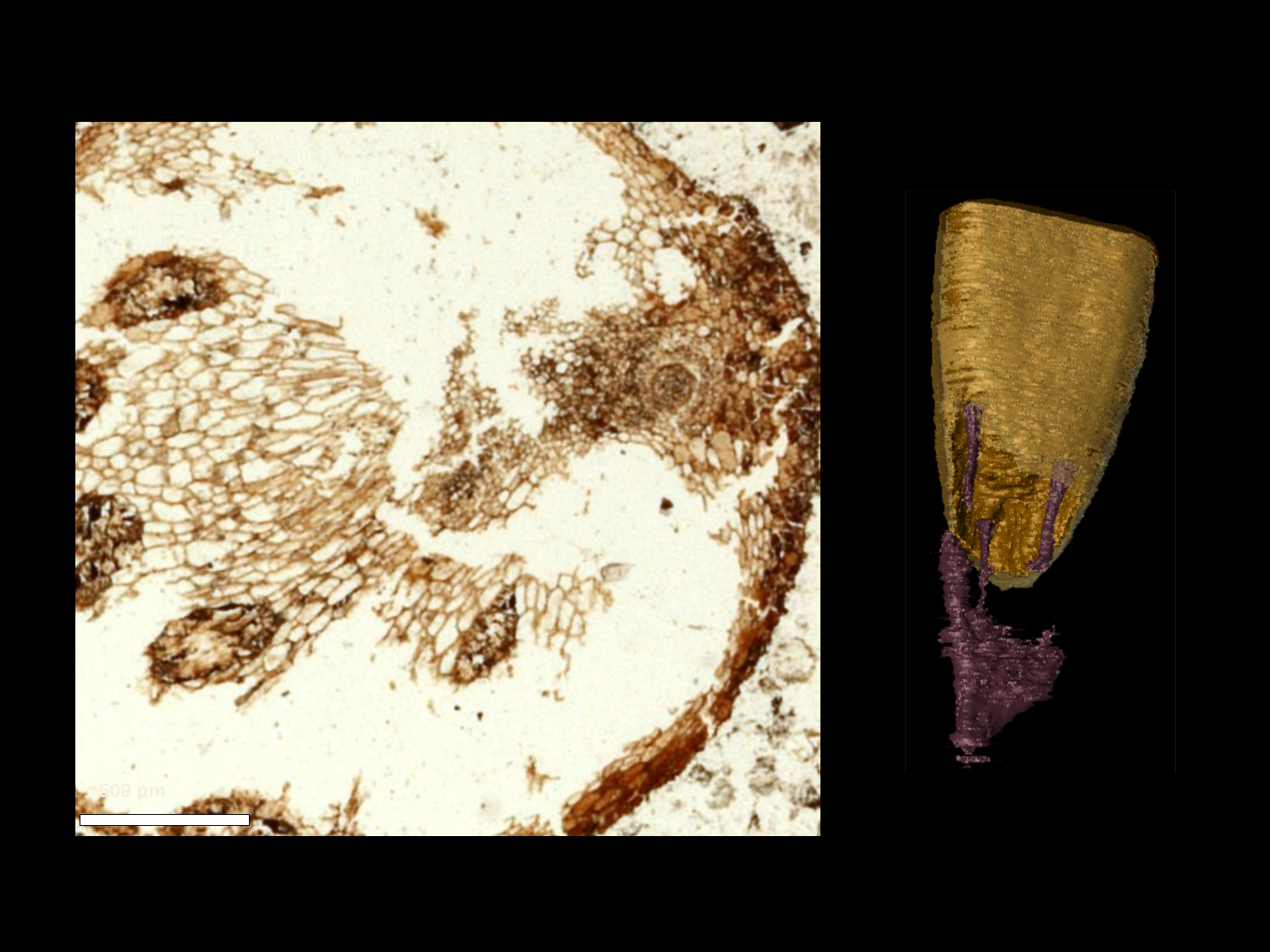

500 μm

## Slide 4
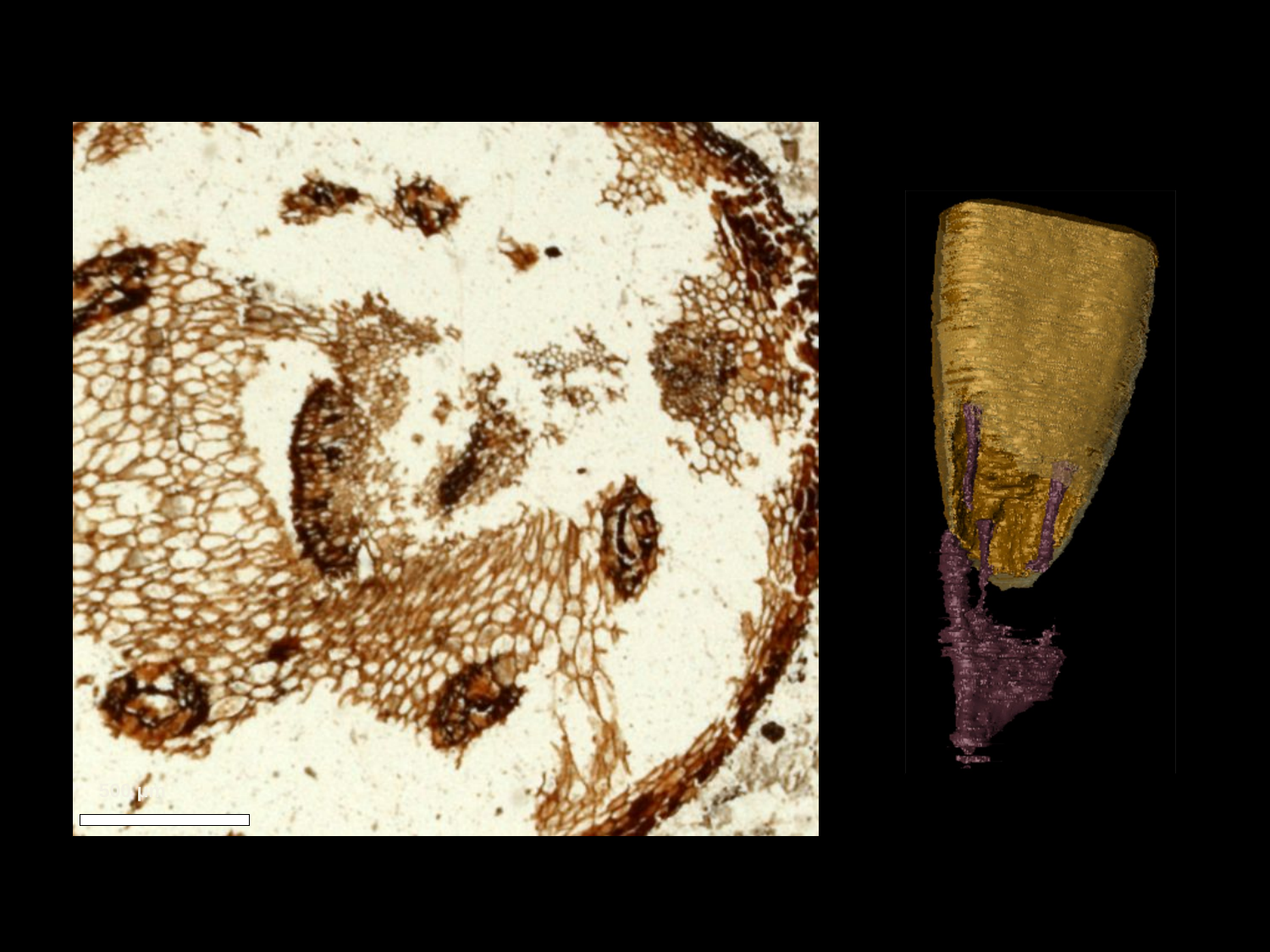

500 μm

## Slide 5
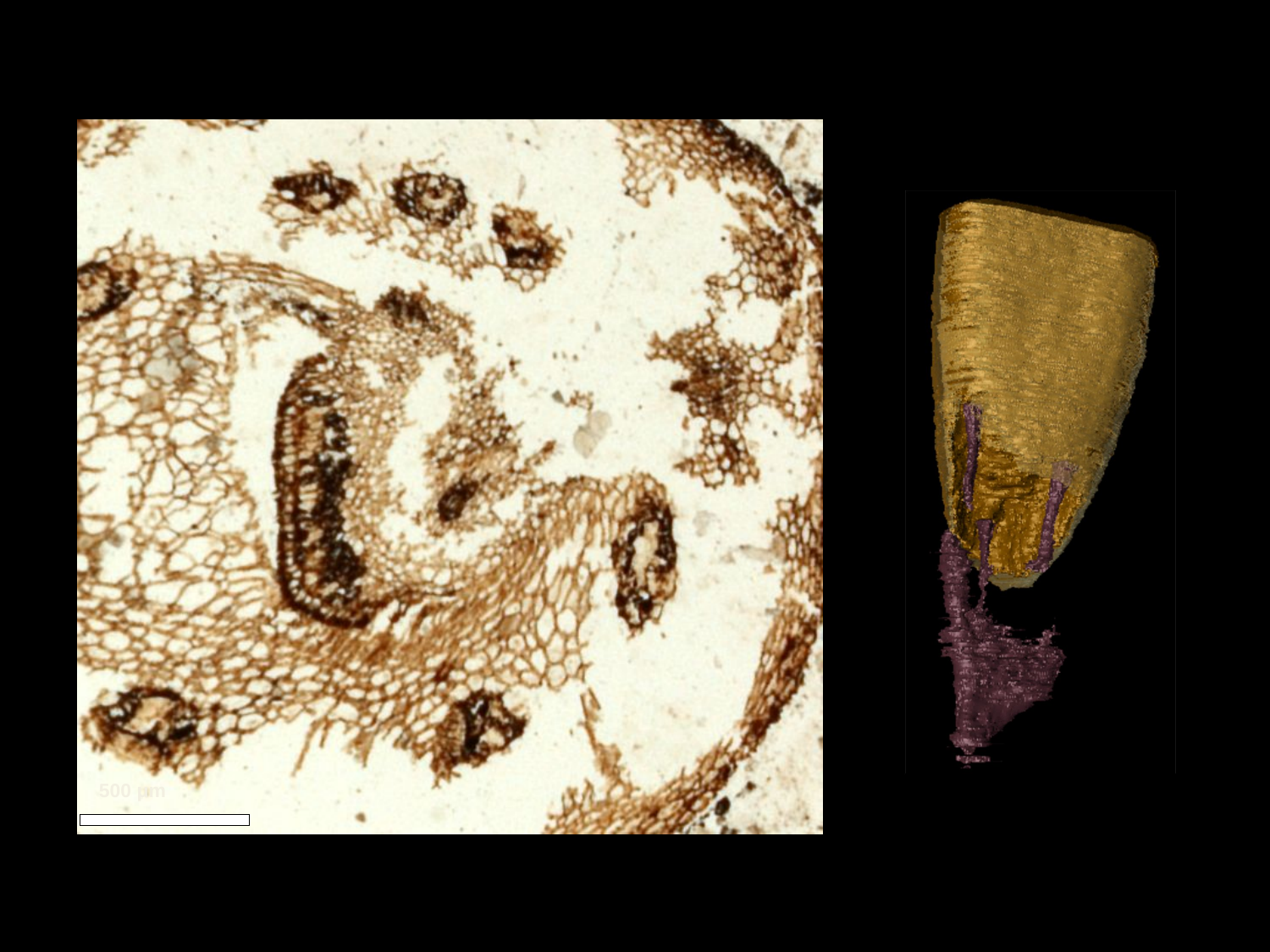

500 μm

## Slide 6
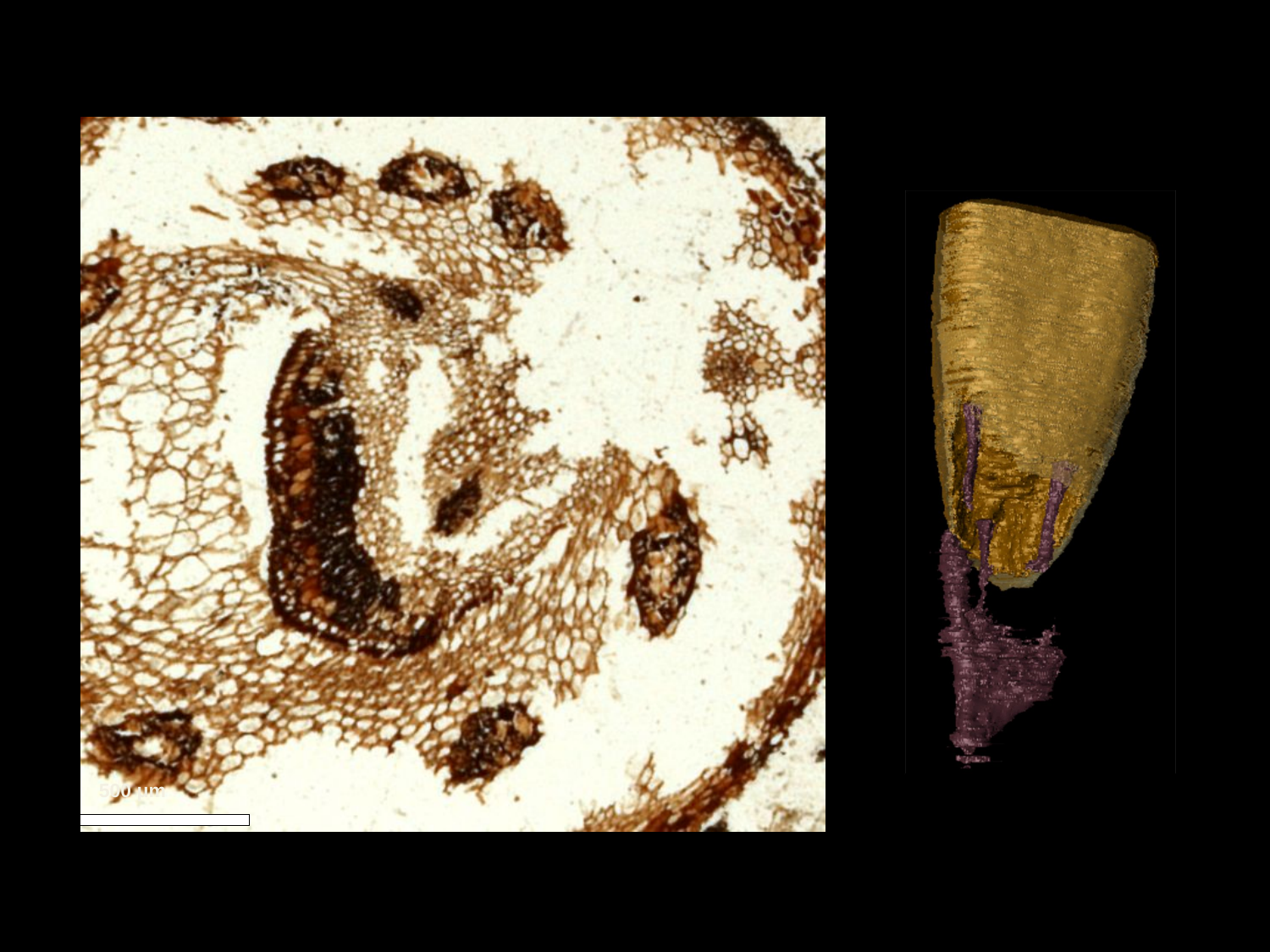

500 μm

## Slide 7
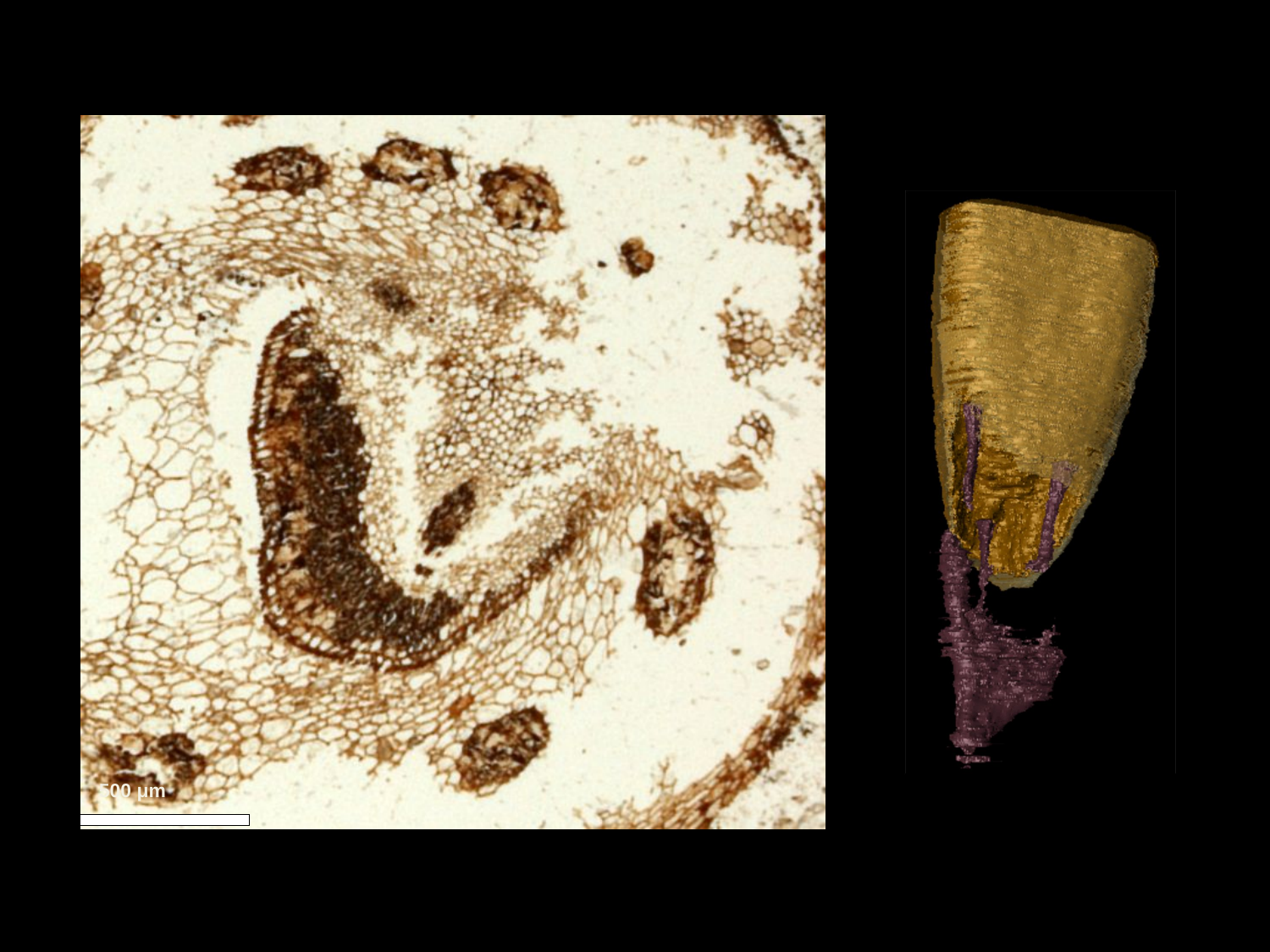

500 μm

## Slide 8
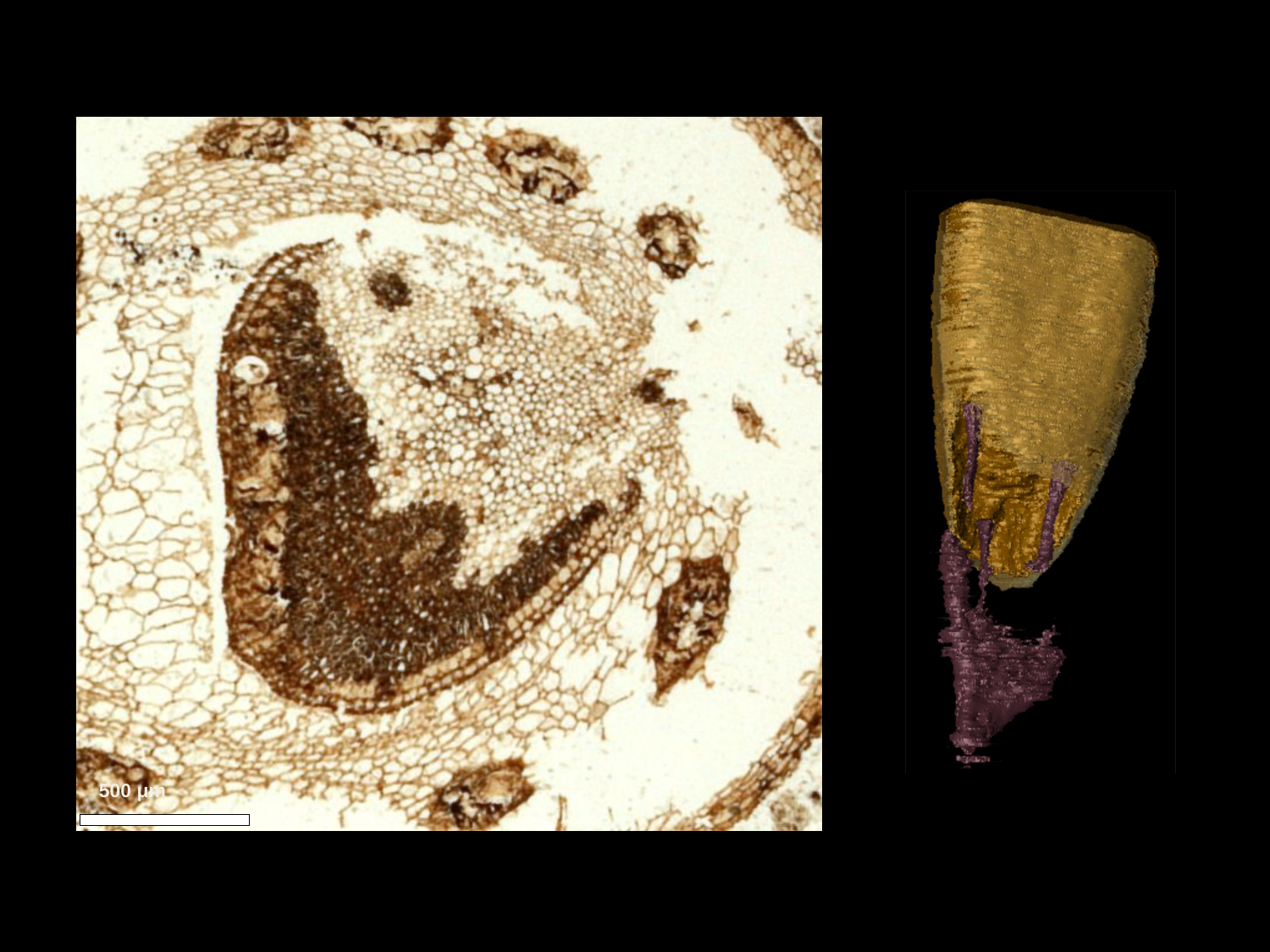

500 μm

## Slide 9
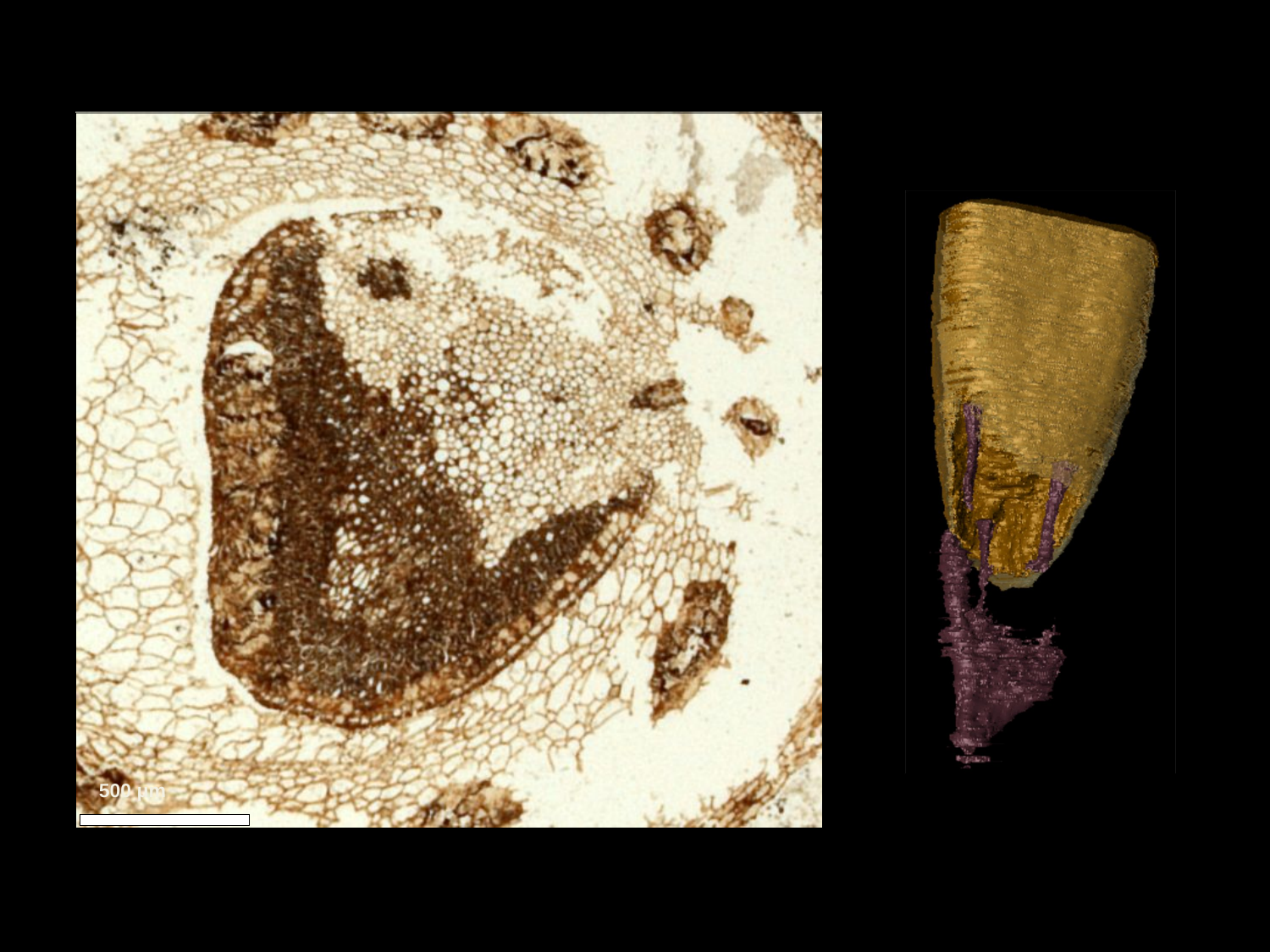

500 μm

## Slide 10
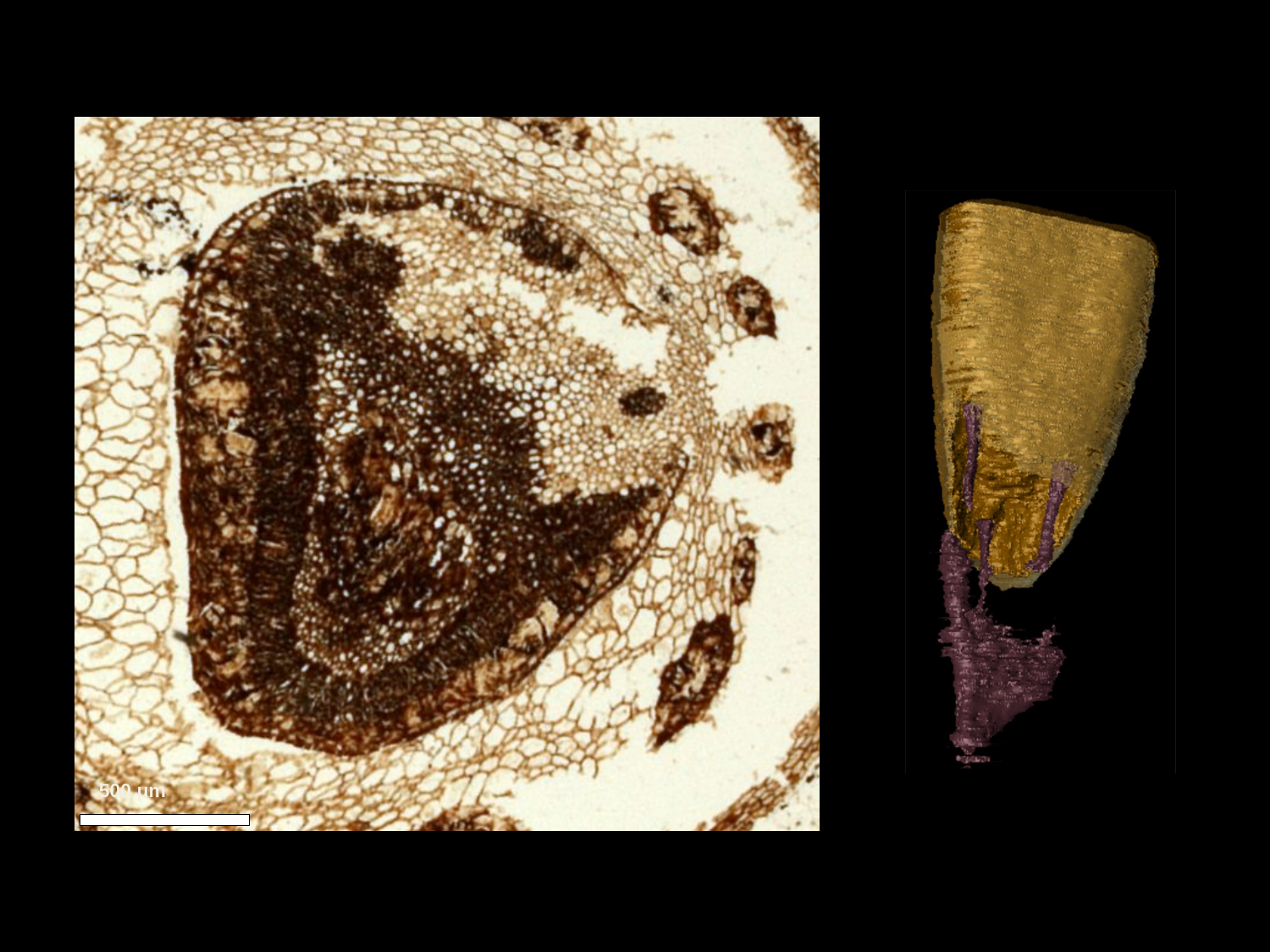

500 μm

## Slide 11
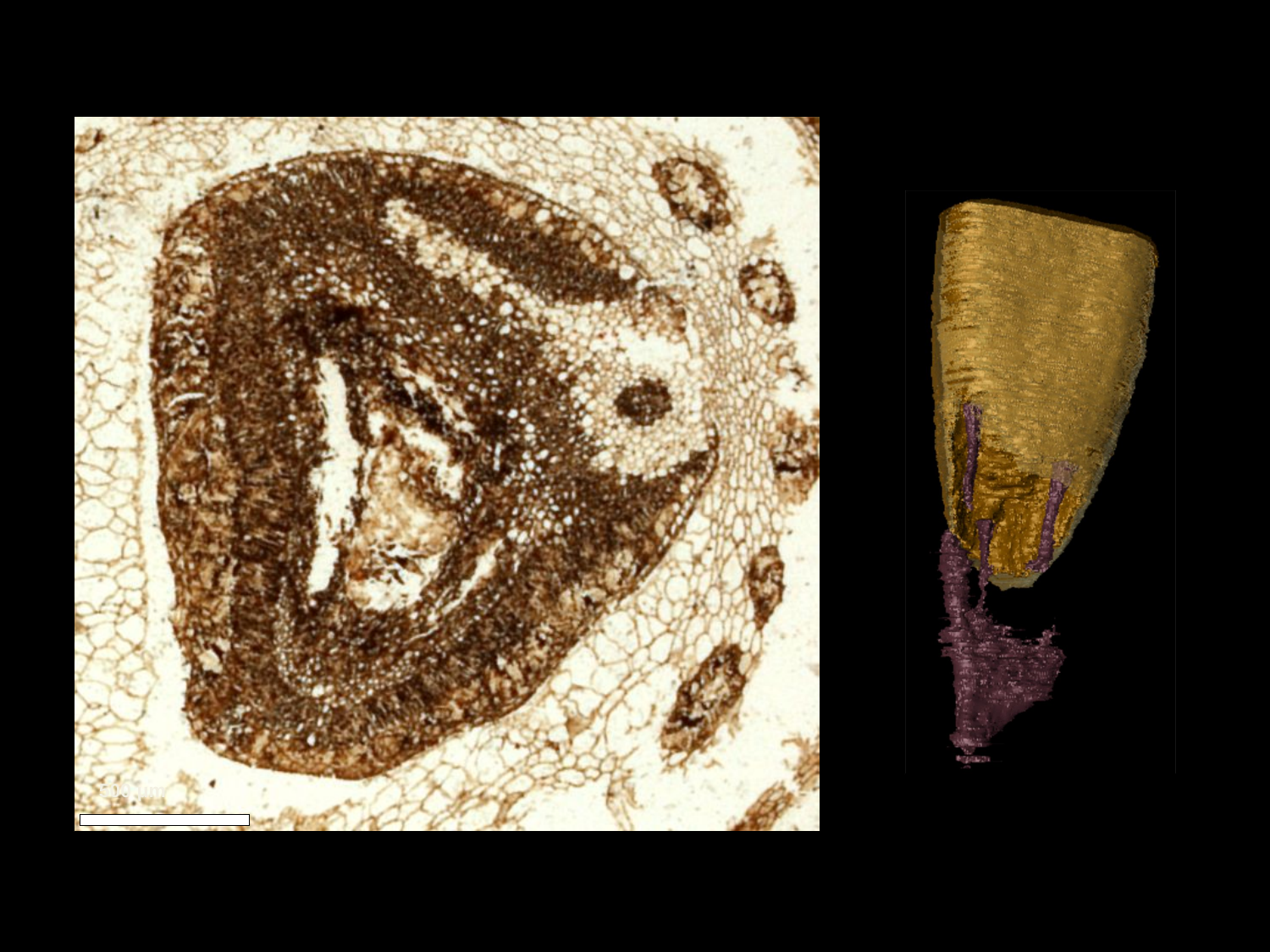

500 μm

## Slide 12
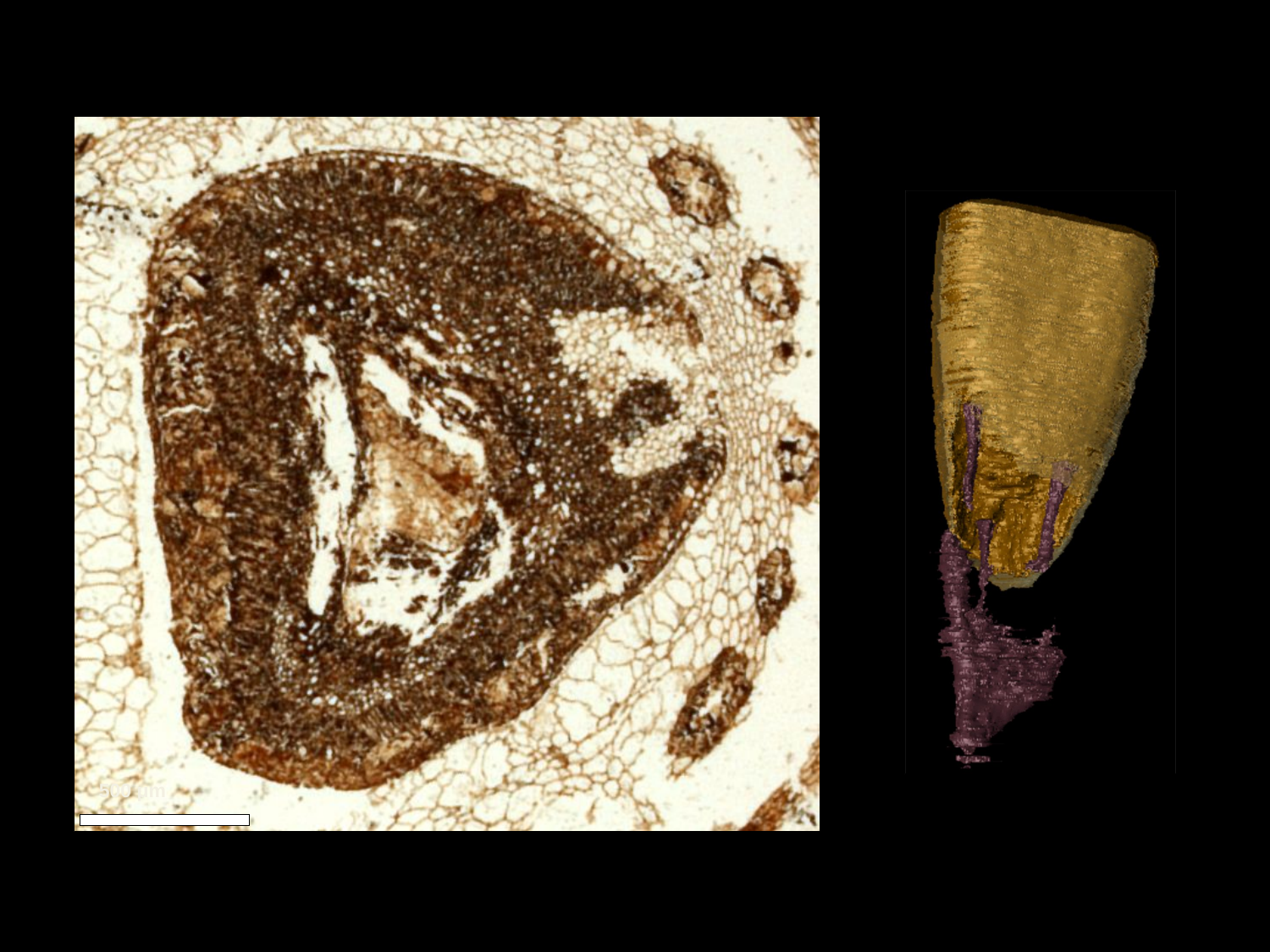

500 μm

## Slide 13
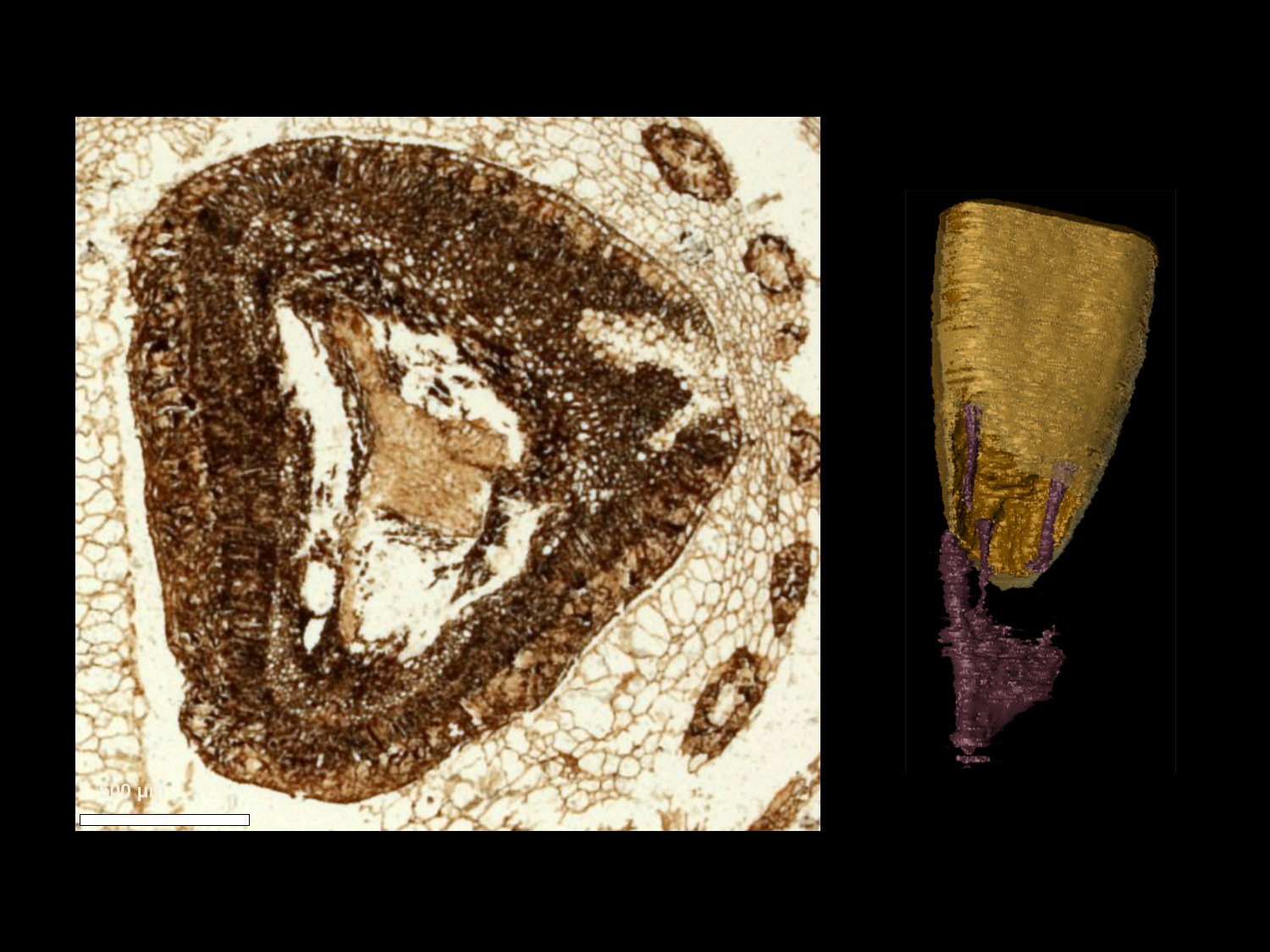

500 μm

## Slide 14
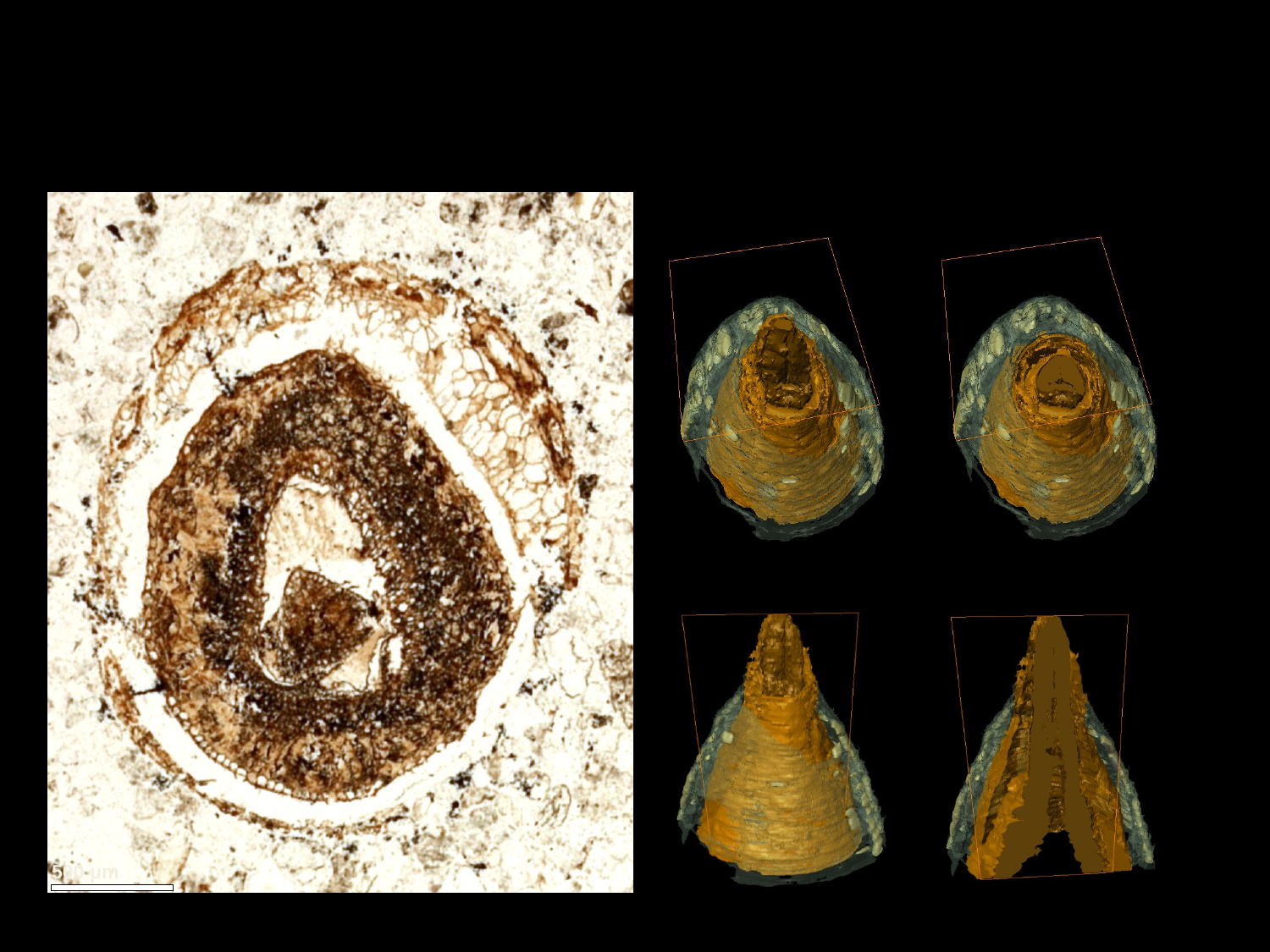

500 μm

## Slide 15
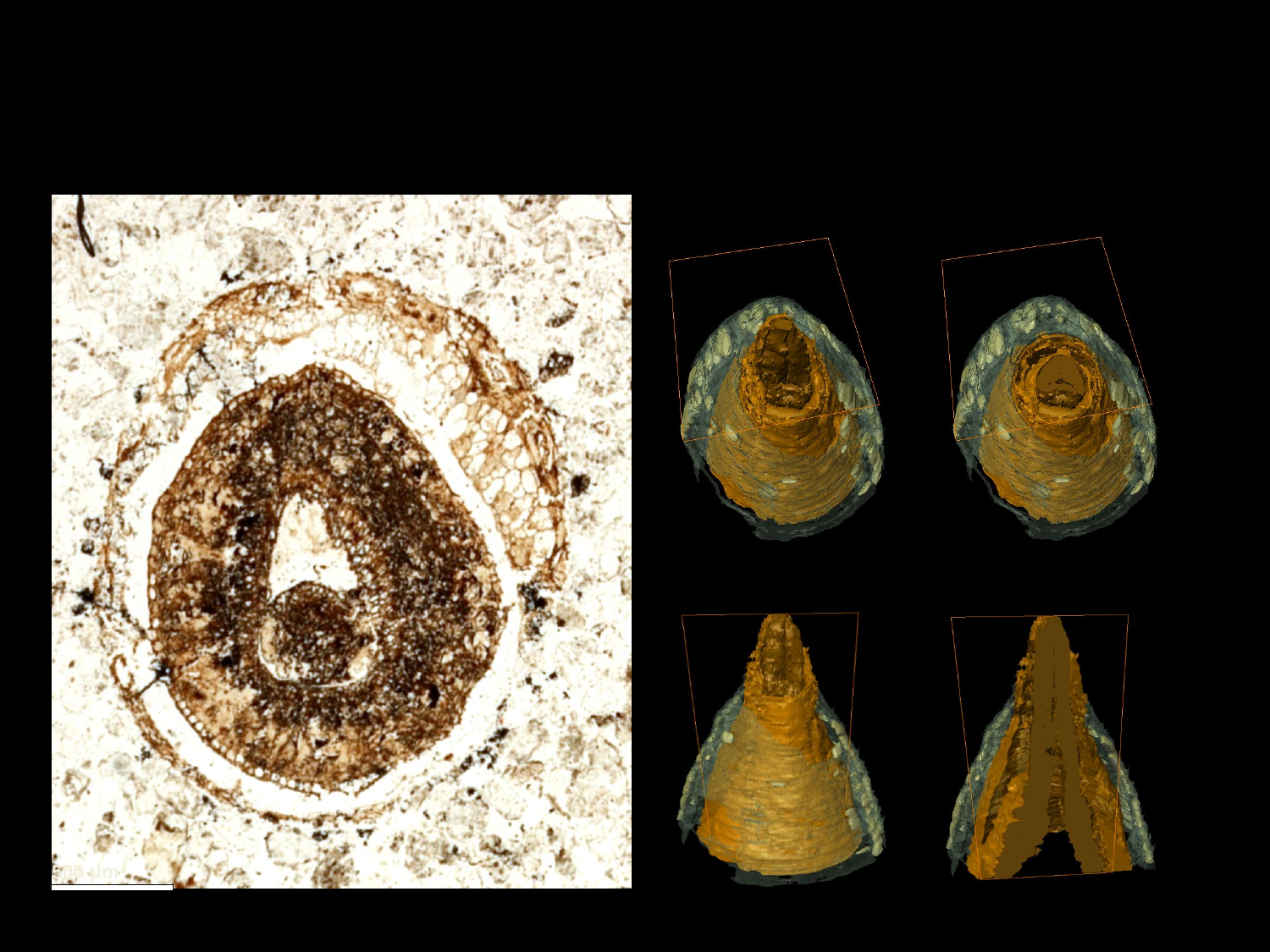

500 μm

## Slide 16
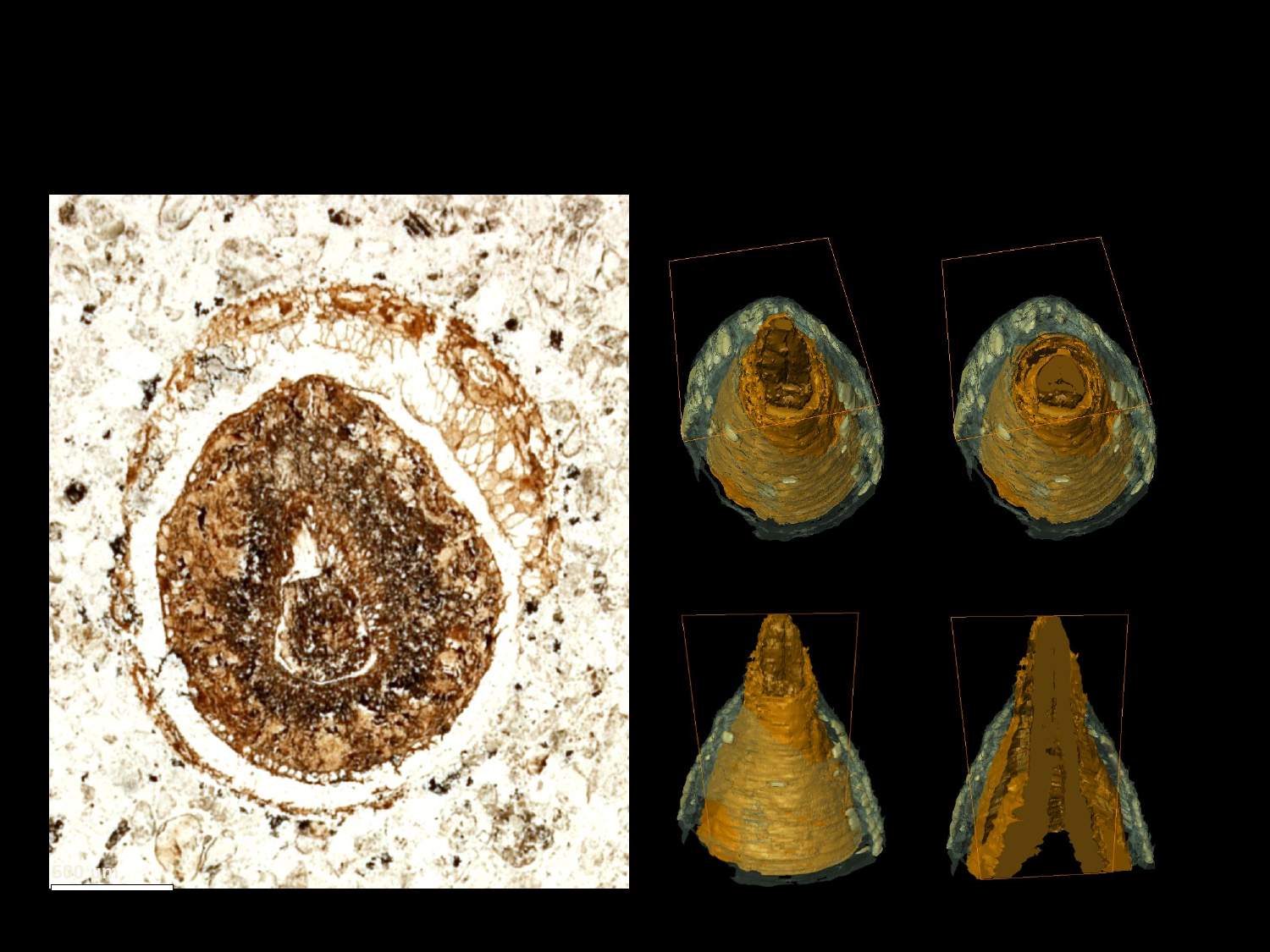

500 μm

## Slide 17
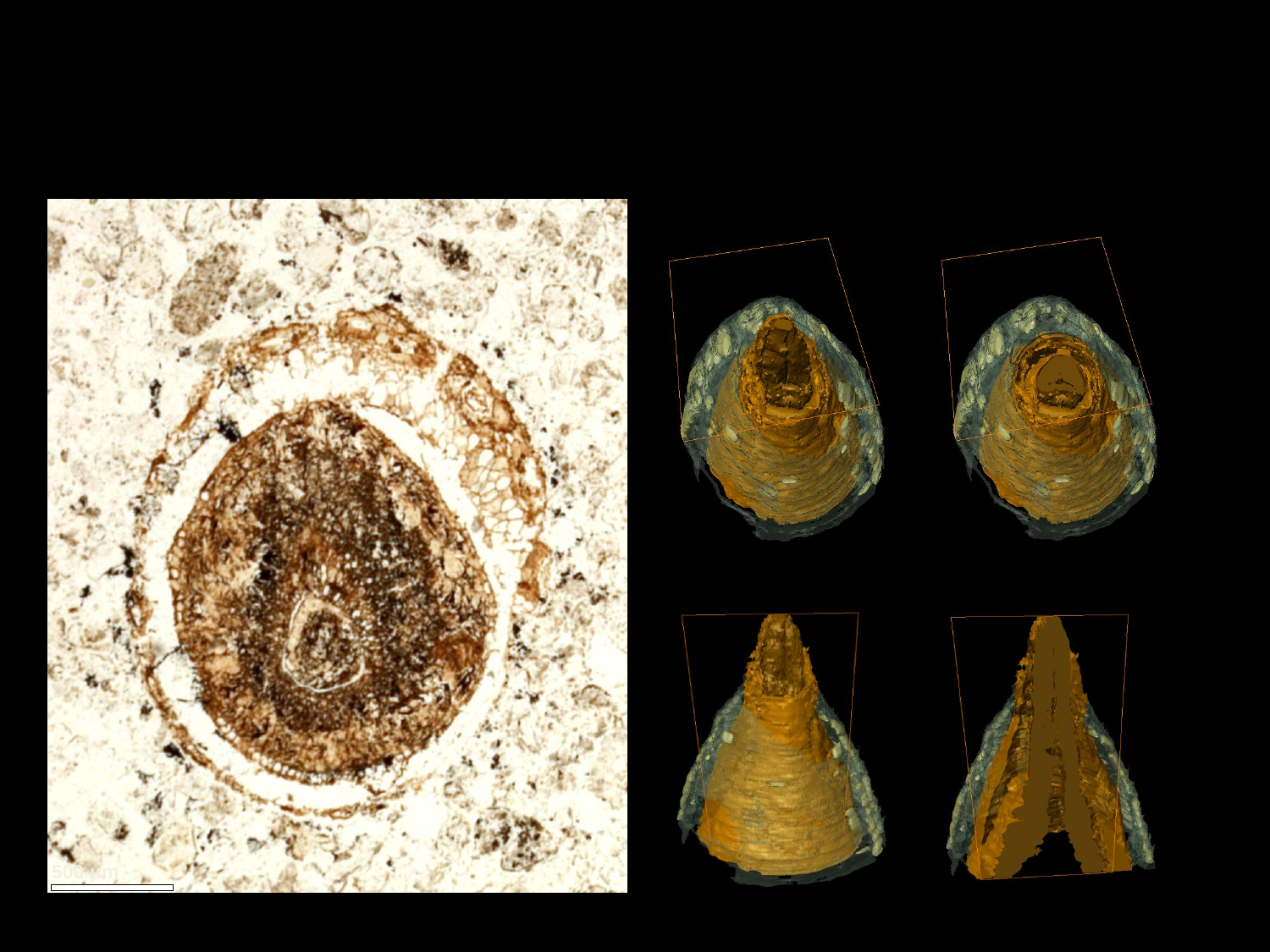

500 μm

## Slide 18
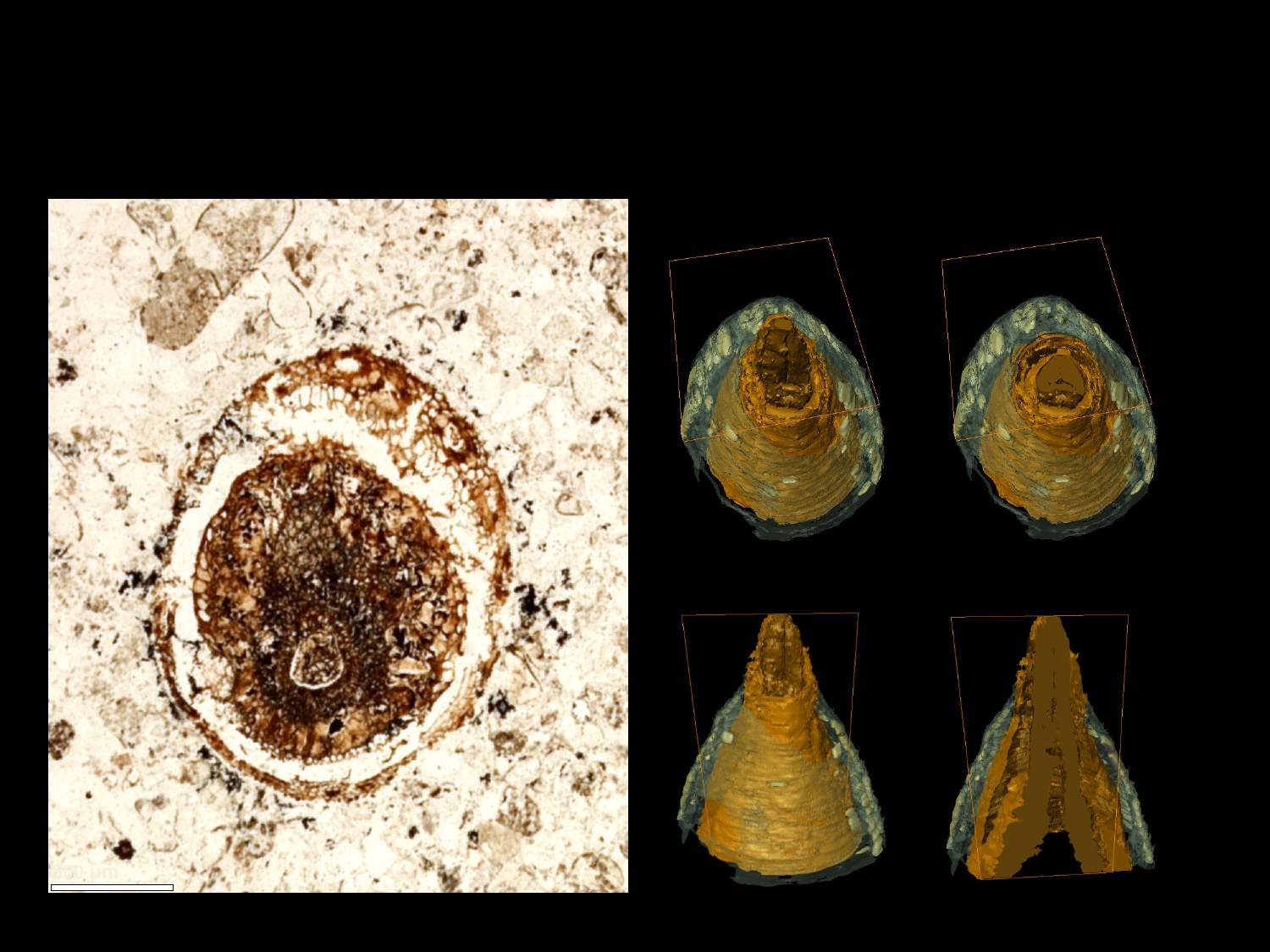

500 μm

## Slide 19
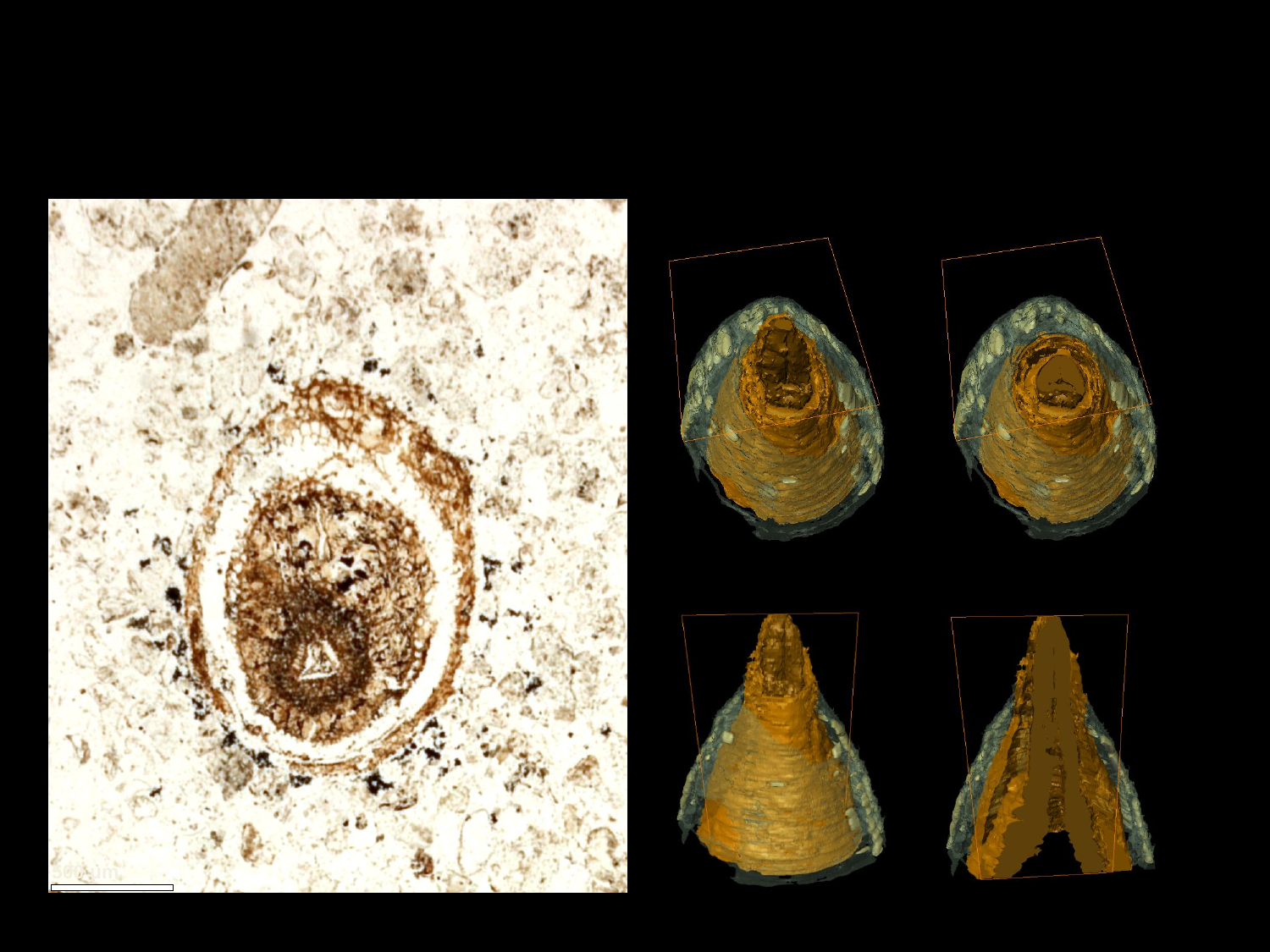

500 μm

## Slide 20
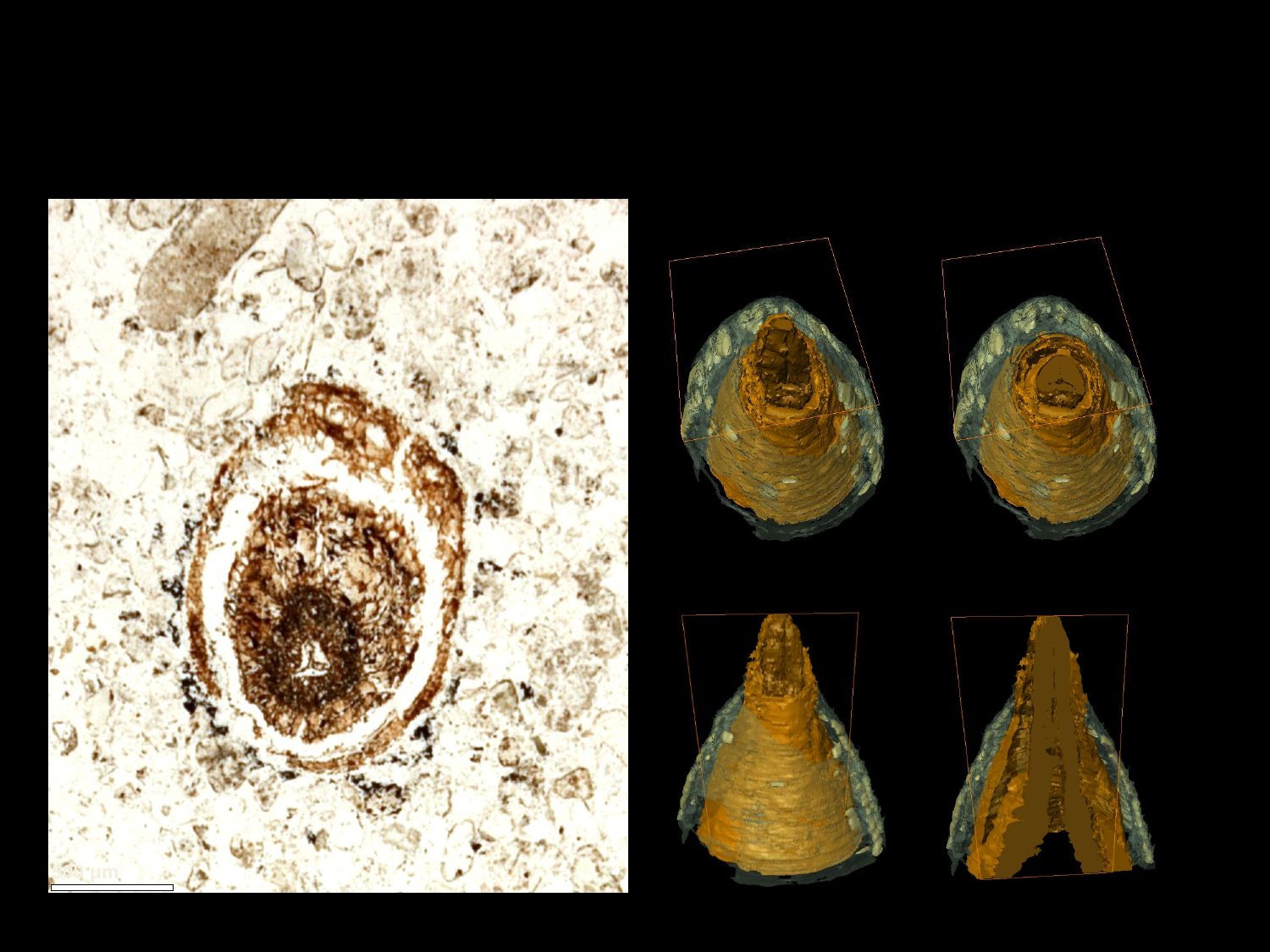

500 μm

## Slide 21
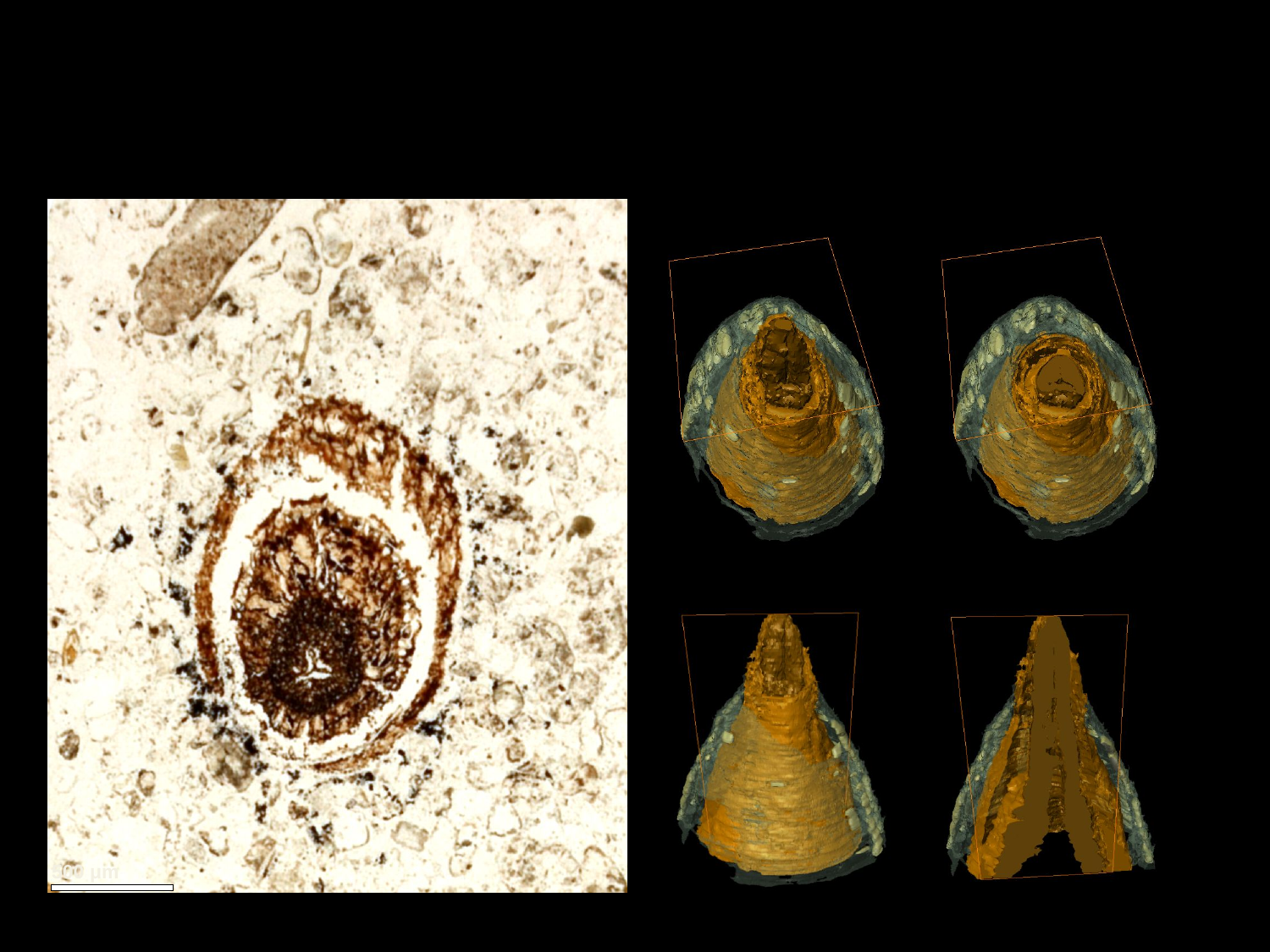

500 μm

## Slide 22
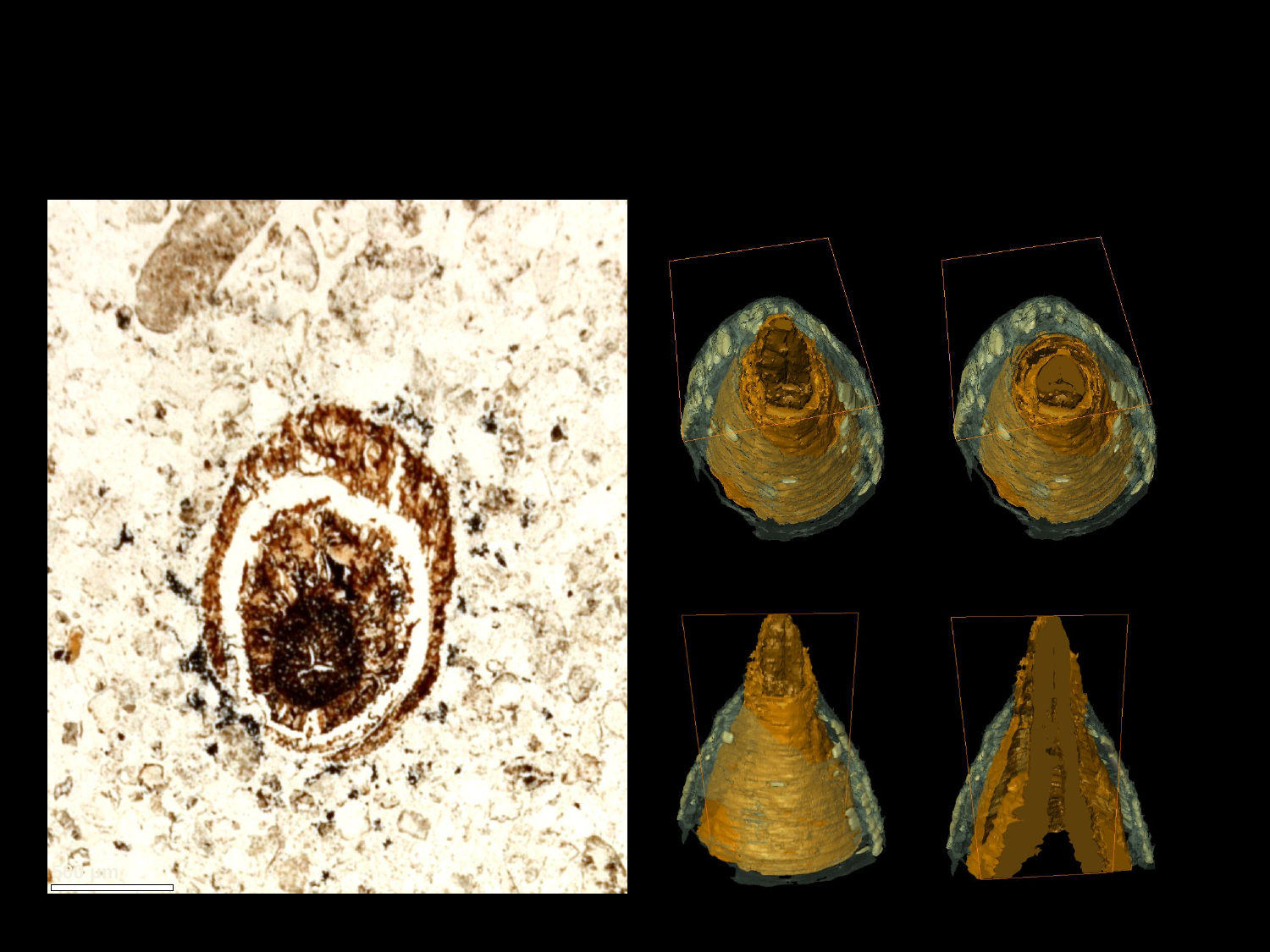

500 μm

## Slide 23
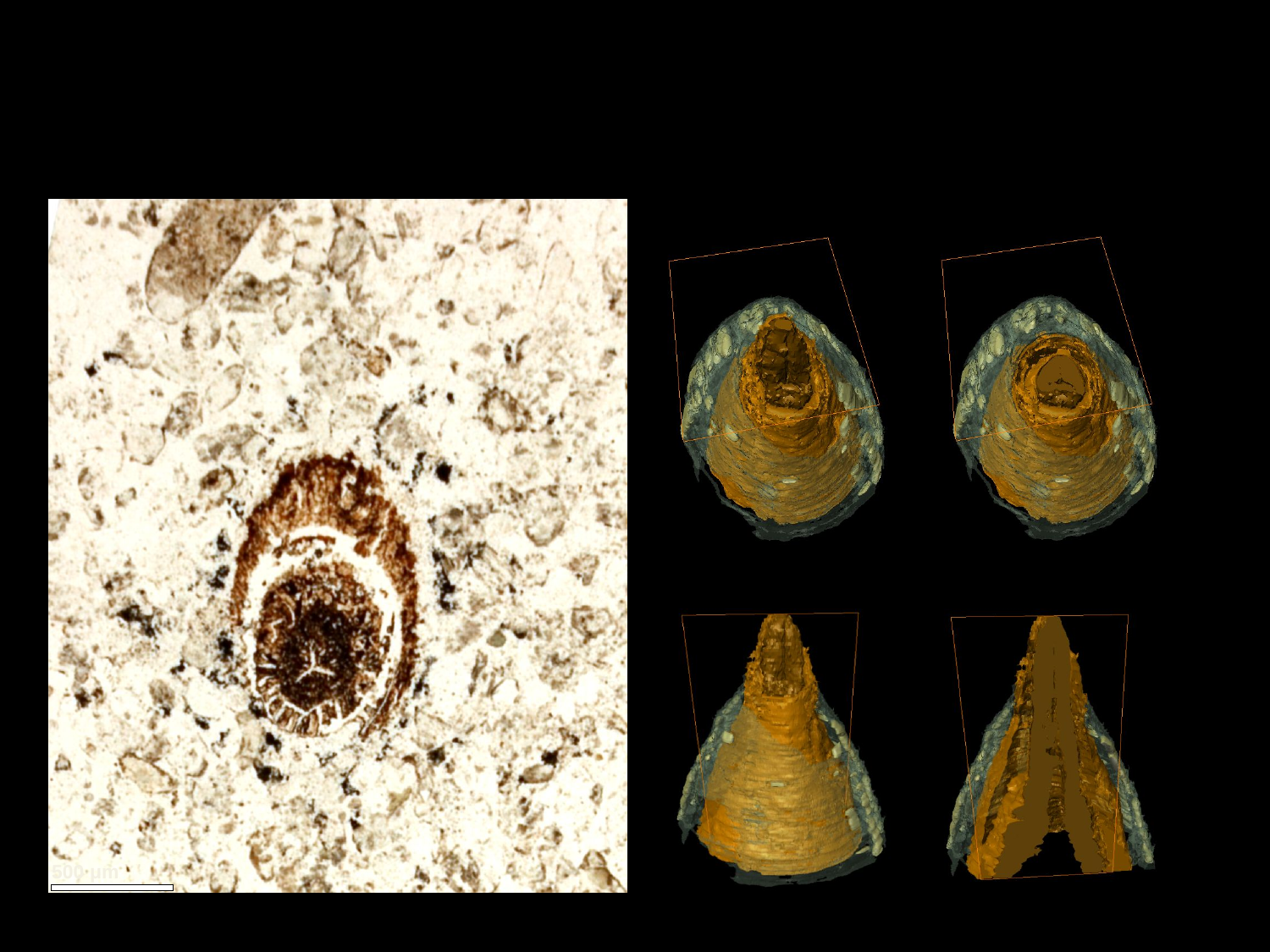

500 μm
